# Reprocessable Networks from Vegetable Oils, Salts, and Food Acids: A Green Polymer Outreach Demonstration for Middle School Students

**DOI:** 10.1021/acs.jchemed.3c01258

**Published:** 2024-06-05

**Authors:** Sara Valdez, Carmen B. Dunn, Miya D. Hullum, Evains Harper, Zhe Qiang

**Affiliations:** † School of Polymer Science and Engineering, The University of Southern Mississippi, 118 College Drive, Hattiesburg, Mississippi 39406, United States; ‡ Hattiesburg High School, 301 North Hutchinson Avenue, Hattiesburg, Mississippi 39401, United States; § N.R. Burger Middle School, 174 W.S.F. Tatum Blvd., Hattiesburg, Mississippi 39401, United States

**Keywords:** Elementary, Middle School Science, Polymer Chemistry, Public Understanding, Outreach, Green Chemistry, Materials Science

## Abstract

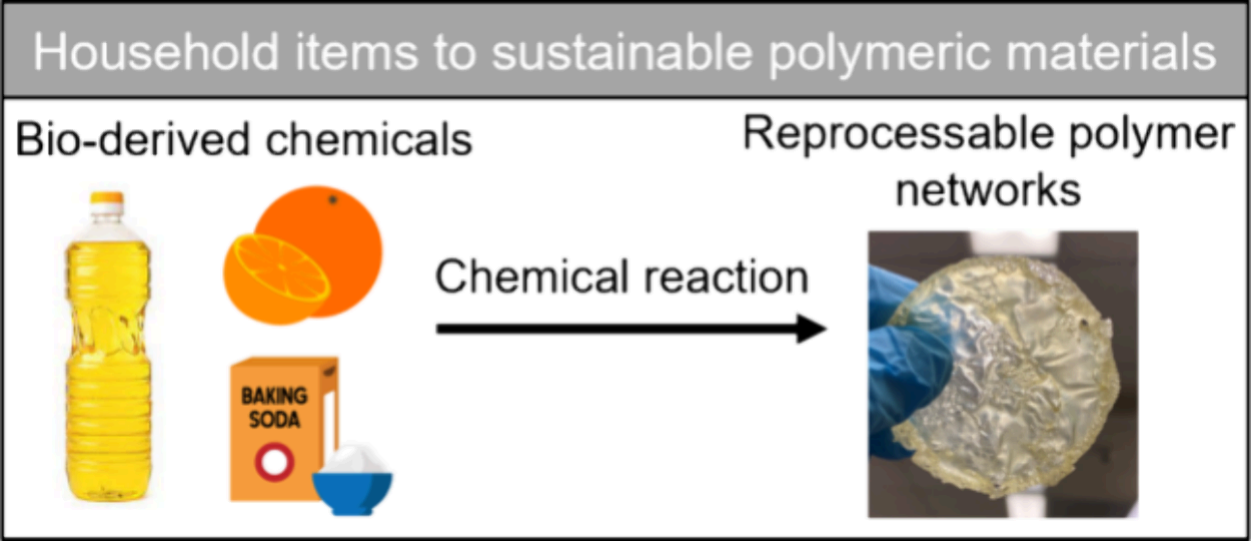

Massive amounts of mismanaged plastic waste have led to growing concerns about their adverse impacts on the environment, ecosystem, and human health. Enabling efficient plastic recycling is a key component for developing a sustainable future, which requires cohesive efforts in technology innovations, public awareness, and workforce development. Particularly, outreach activities to inform the broader community about current efforts to fabricate sustainable polymeric materials can play a central role in inspiring future generations while also improving their knowledge, viewpoints, and behaviors to address plastic waste challenges. Herein, this account demonstrates an effort to educate middle school students about a key emerging concept in polymer science for sustainable material development: reprocessable polymer networks. Background information is provided to the students about the need to transition from petroleum-based chemical feedstocks to their bioderived counterparts. We note that the materials used in this demonstration lesson are all produced from common household foods, with which students routinely interact in various applications, making them not only safe but also compelling for the middle school classroom.

## Introduction

Polymer materials have a ubiquitous role in our everyday lives due to their low cost, ease of production, and ability to access a broad range of different properties.^1^ The annual production volume of plastics has reached nearly 500 million metric tons. However, most of them are mismanaged at their end of life, including being incinerated, landfilled, and improperly discarded, which results in pollution to the environment that is becoming significantly detrimental to human health, aquatic life, and the climate.^2−4^ To address these challenges, the plastic industry is experiencing an exciting transition from a linear to circular system, of which recycling is a crucial component. Collaborative efforts from the government, industry, and academia have made significant contributions in research and outreach which provides an exciting opportunity to educate future generations about the design and need of sustainable polymer materials.^5^

In general, there are two major types of polymers, including thermoplastics and thermosets.^6^ The key difference between them is associated with processability (i.e., describing the ability of them to transform from materials to products). Specifically, thermoplastics can flow at elevated temperatures enabling them to be engineered into different products, while thermosets are crosslinked and maintain a permanent shape. The crosslinks are covalent bonds that interconnect different polymer chains into a network, making thermosets very challenging to be (re)processed into new materials and products. Currently, thermoplastics are significantly easier to recycle at a large scale. Commercially, the majority of recycling processes taking place is mechanical recycling (melting/reprocessing into a new product). However, it is best suited for single-plastic waste streams that are extremely difficult to obtain.^7^ Chemical recycling (e.g., depolymerization) is another option, but it is used far less frequently on a commercial level due to its high energy requirements and costs. As a result, the recycling of thermosets is an intractable challenge in the plastic industry.^8^

In recent years, extensive research efforts have been focused on the development of reprocessable networks as an alternative to conventional thermosets. These materials contain crosslinks that can be dynamic (or rearranged) under specific conditions (different external stimuli), such as heat or light, enabling simultaneous crosslinking features and processability, which hold great potential for various applications including self-healing coatings, stimuli-responsive materials, and recyclable composites.^9^ Moreover, an important aspect in designing new materials is how the process aligns with the principles of Green Chemistry,^10,11^ which is defined as the “design of chemical products and processes to reduce or eliminate the use and generation of hazardous substances”. Specifically, the need to transition from petroleum-based systems to bioderived and renewable resources is strong, providing potential opportunity to reduce carbon emissions and other environmental impacts from current plastic manufacturing activities.

This paper describes a simple demonstration to middle school students about the concept of reprocessable polymer networks using household food-relevant resources, wherein current educational literature for reprocessable networks primarily focuses on undergraduate laboratory experiments/research and not K–12 outreach.^12,13^ This demonstration can be accomplished over two 50 min class periods. Specifically, the first class focuses on introducing fundamental concepts about polymers, their recycling challenges, and their environmental impacts. In the second class, hands-on demonstrations are provided to show differences in conventional polymer networks and their reprocessable counterparts that are derived from food grade acids, vegetable oils, and salts. Through these engaging activities, students can increase their knowledge of chemistry and polymer science while becoming more aware and inspired about the opportunity to address the plastic waste challenge through science and technology.

## Experimental Section

### Materials and Consumables

The necessary materials and equipment for the demonstration are listed below:

- Epoxidized soybean oil (ESO)
- Citric acid
- Sodium bicarbonate (i.e., baking soda)
- Ethanol
- Water (tap water is suitable for demonstration purposes)
- Glass Petri dishes
- ∼500 g weight
- Aluminum weigh boats, glass vials, pipettes, a razor blade, gloves, and a hot plate.

The soybean oil used was obtained from Thermo Scientific and was epoxidized with meta-chloroperoxybenzoic acid (mCPBA, described in Supporting Information) before synthesizing the polymer networks. This step is accomplished in the lab prior to visiting middle school classrooms. We note that epoxidized soybean oil is also commercially available. Ethanol (200 proof) was obtained from Fischer Chemical, and citric acid and sodium bicarbonate were obtained from Sigma-Aldrich; all were used as received.

### Procedures for Material Synthesis

**Figure 1:**
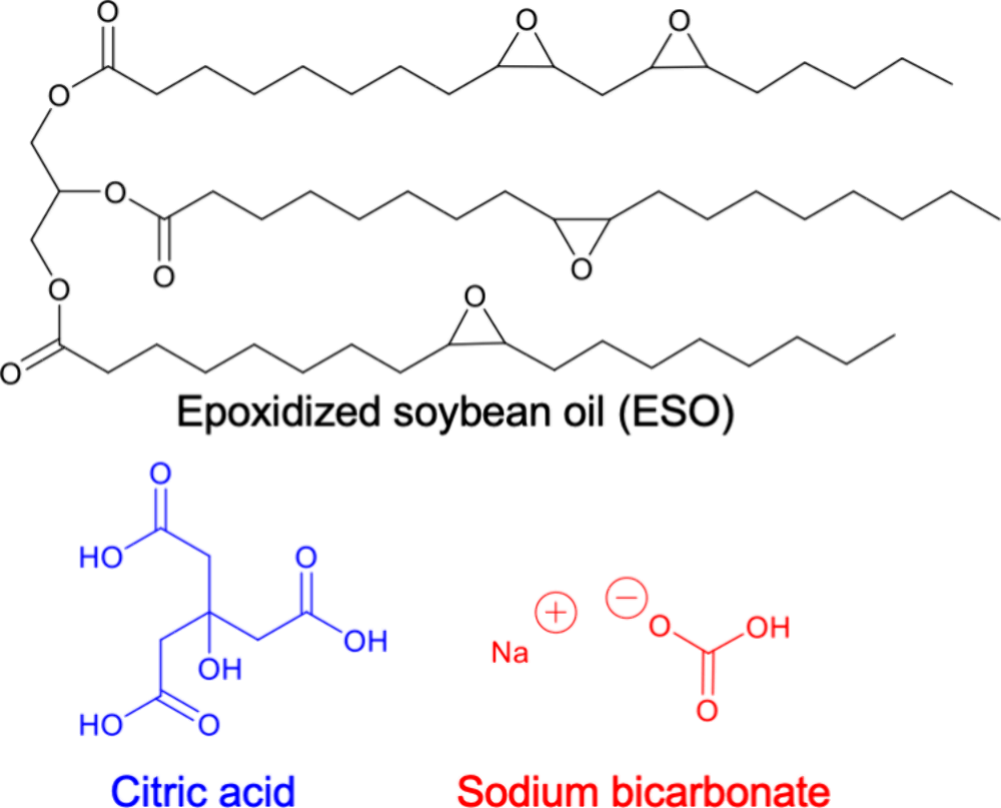
Chemical structures of the reagents used in the formation of the networks.

Both the thermoset and the reprocessable network are based on the esterification reaction between the epoxide groups in epoxidized soybean oil (ESO) and the carboxylic acid groups of citric acid. The chemical structure of compounds used in this demonstration are shown in Figure 1.

To prepare the thermoset, ESO (5.0 g, 5.07 mmol, 1 equiv) and citric acid (1.3 g, 6.74 mmol, 1.33 equiv) were weighed into separate glass vials with a 1:1 epoxide/carboxylic acid ratio. Subsequently, a small amount of ethanol (∼5 mL) was added to the vials for dissolving the ESO and citric acid. The contents of the vial were then poured into an aluminum weigh boat for evaporation. After removal of the ethanol, the reaction mixture was heated on a hot plate at 100 °C for 30 min and then at 125 °C for 45 min for curing. A similar procedure was employed for preparing reprocessable networks, with the addition of sodium bicarbonate (63 mg, 1 wt %) to allow reprocessing to occur. Fourier transform infrared spectroscopy (FTIR) was used to confirm the formation of the materials during the design of the demonstration but is not necessary for future instructors; the reported procedure is reproducible, and the results can be found in the Supporting Information.

### Demonstrating Reprocessability

To demonstrate reprocessability, two pieces of each network (∼1.3 cm squares) were cut from the samples using a razor blade and placed on the top of a glass Petri dish. The pieces from each network were then stacked, partially overlapping. The bottom of the Petri dish was then placed on top of the dish to give a flat surface for adding the weight. The Petri dish was then moved to a hot plate set to 125 °C with the 500 g weight on top for approximately 10 min. Subsequently, the weight was removed, and samples were naturally cooled to room temperature. Students were then allowed to come forward and look at the two networks up close, as well as put on gloves and touch samples of both networks to further understand their differences. Detailed procedures for material preparation are included in the Supporting Information.

## Safety and Hazards

Personal protective equipment must be worn when performing the demonstration (including a lab coat, safety glasses, gloves, and closed toe shoes). During heating with the hot plate, attention should be paid due to elevated temperatures and heat gloves should be worn. Epoxidized soybean oil is slightly irritating to the eyes and skin. Citric acid should be used in a well-ventilated area, and ethanol is flammable and should not be used around an open flame. It is also recommended that extra caution be used when handling the razor blade to avoid cuts.

## Results and Discussion

### Rationale of Chemical Selections

Vegetable oils are triglycerides extracted from various plants and have been used in food and cosmetic applications for centuries. In recent years, vegetable oils have gained significant attention in academic research and industrial applications due to their low cost, renewability, biodegradability, abundance, and versatility. Specifically, vegetable oil-based biofuels have become a promising renewable energy source.^14^ In this demonstration, soybean oil was selected due to its prevalence, as it is one of the most widely produced vegetable oils, only second to palm oil, with over 40 million metric tons produced worldwide each year for the past decade.^15^ Moreover, this raw material is abundant in the Southern regions of the U.S., which can make Mississippi students feel more relevant and engaged.

Citric acid, the other main component of the networks (crosslinker), is an organic tricarboxylic acid found in a variety of citrus fruit juices and pineapples that has broad applications from a vitamin preservative to use in the detergent industry as a substitute for phosphates.^16^ We note citric acid is environmentally friendly and can be commercially produced by different microorganisms (e.g., bacteria, fungi, and yeast).^17^

Baking soda, the catalyst added to the reprocessable networks, is a water-soluble acid salt of sodium and bicarbonate with a wide range of applications, including cleaning, fire extinguishing, deodorizing, and baking. Baking soda is made from soda ash (sodium carbonate) which can be prepared using the Solvay process or from mined trona ore.^18^

**Figure 2:**
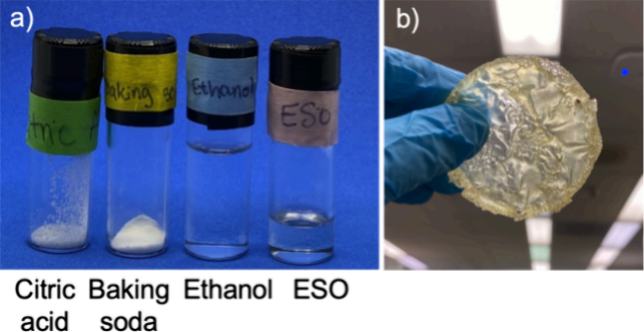
(a) Starting reagents and solvent for the networks and (b) cured reprocessable network.

We note that these three reagents (all shown in Figure 2a) were selected because it is very likely that most students have already interacted with them, such as in their home kitchens. It is important to recognize that using bioderived materials does not necessarily mean they are more sustainable and/or “greener” than their petroleum-derived counterparts.^17^ However, for the purpose of outreaching, starting with materials that they are familiar with, instead of other “unfamiliar chemicals”, helps the demonstration and concept be easier to follow and grasp. Additionally, using reagents that can be found in nature or are biorenewable helps to address and make connections with socioscientific issues such as sustainability, that can inspire the students to have improved critical thinking skills and greater awareness of the world around them.^19^

Figure 2b shows a cured, reprocessable network sample. After heating, both types of samples were relatively translucent (sample roughness caused opacity in some samples) and the thermosets tended to be more yellow in color, while the color of the reprocessable networks was a pale yellow.

### Mechanisms of Cross-Linking and Reprocessability

**Figure 3:**
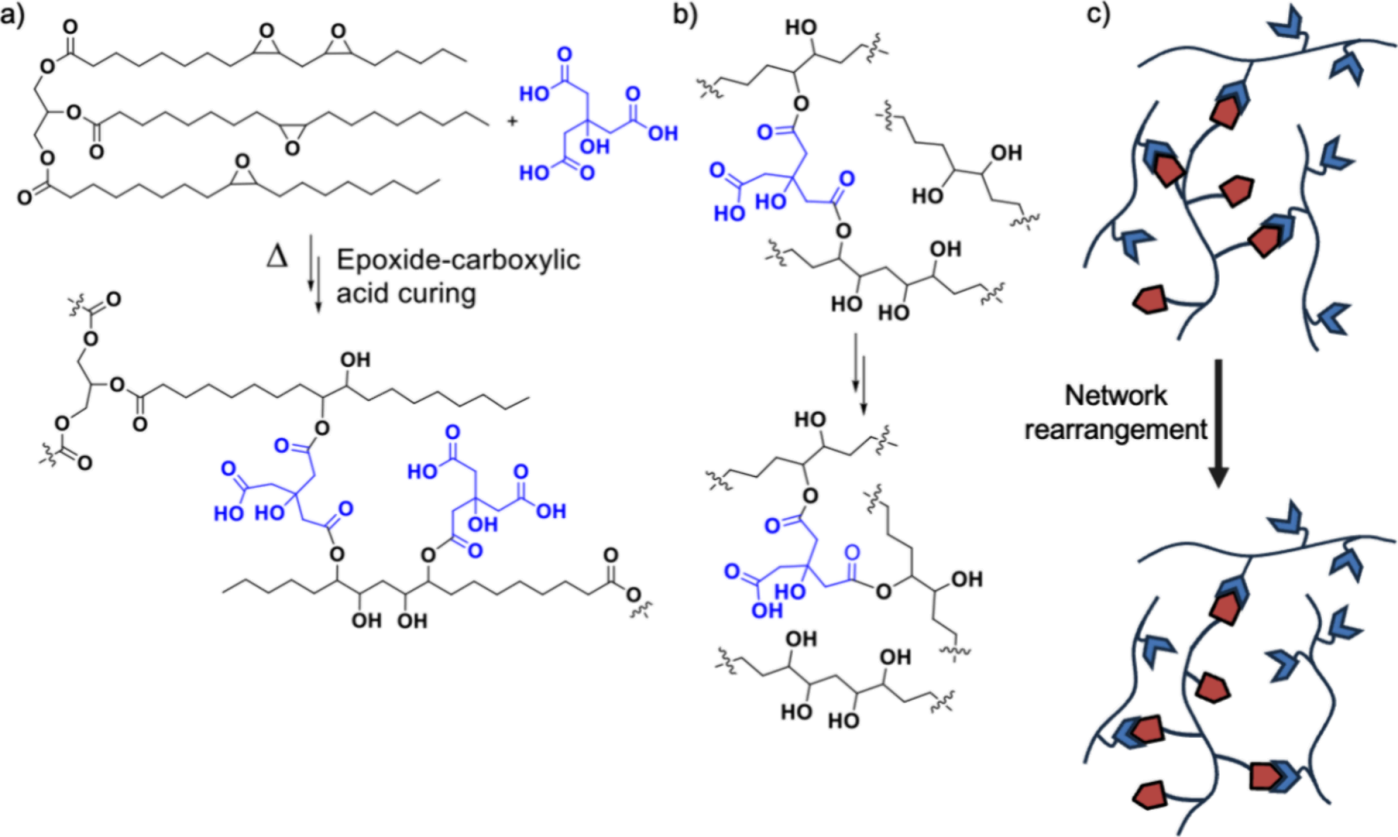
(a) Reaction scheme of thermoset formation, (b) transesterification of the reprocessable network, and (c) graphic showing reprocessability.

The reaction scheme of network formation and transesterification can be found in Figure 3. From a fundamental chemistry perspective (information for teachers), Figure 3b shows the transesterification between the initially formed esters between the citric acid/epoxide (Figure 3a) and the free alcohols formed after epoxide ring-opening. This reaction enables the reprocessable network to rearrange and flow with the application of heat and pressure. However, transesterification will not occur under neutral, uncatalyzed conditions, leading to the permanent shape formed in the thermosetting samples. Specifically, baking soda serves as a basic catalyst in this system and causes a two-step addition–elimination process to occur (nucleophilic acyl substitution).

To briefly describe this process in a grade-appropriate manner, by the time students are in the 7th–8th grade, they have been taught what atoms/elements are, so we explained the concept of functional groups as specific arrangements of the atoms that allow reactions to occur. Only certain functional groups can react together under specific conditions and can be considered puzzle pieces that click together (Figure 3c). Once they understand the concept of catalysis, it is easier for them to grasp where the conditions can be made less intense (lower temperatures) for reactions to proceed or make reactions more efficient.

**Figure 4:**
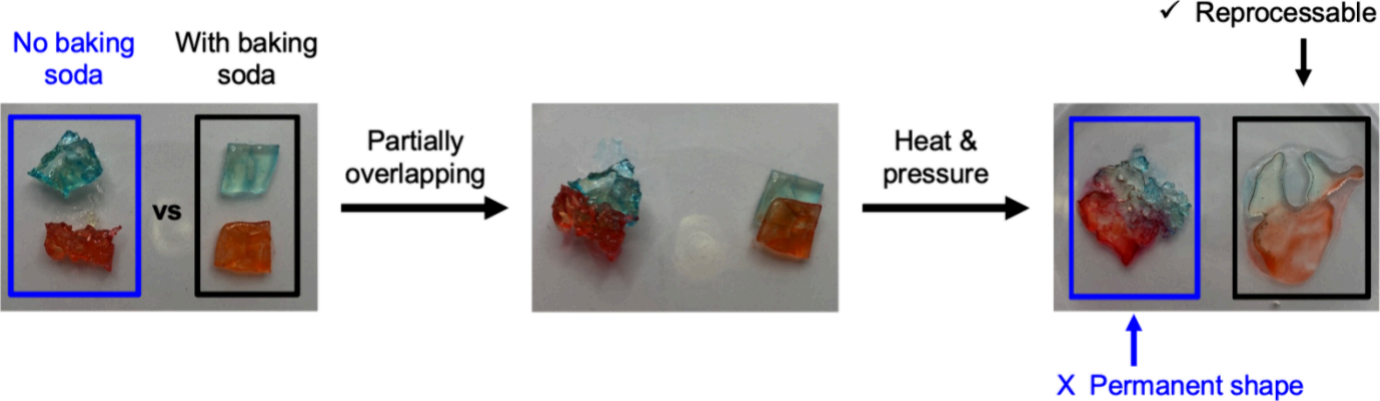
Reprocessability demonstration of the thermoset (left) and reprocessable network (right).

A simple demonstration was done for the class through visualizing the differing nature between a permanently cross-linked network and its reprocessable counterpart. The sample containing baking soda has a rearrangeable network structure and, thus, the ability to flow upon pressure at elevated temperatures. As shown in Figure 4, a sample of each network was first provided for the students to compare their appearances. During demonstrations at the school, the samples were not dyed but are here for clarity, so it is easier to follow the shape changes in the materials. Methyl red and methylene blue hydrates (both obtained from TCI Chemicals) in ethanol were used to dye the sample pieces. Subsequently, both samples were cut in half and stacked partially overlapping, followed by the application of pressure and heat (500 g weight at 125 °C). It can be observed that the reprocessable network can become a continuous network, while the thermoset is still a separate piece that cannot be reformed. Here, students noticed that the reprocessable network has also gotten much flatter because not only is it reformable but also the heat allows the material to flow (confirmed by the flow features of incorporated dyes), while the thermoset is not able to; thus, it keeps a similar shape (and thickness) to its original. This can also be seen in the images in Figure 4, where the thermoset dye concentrations are about the same before heat and pressure are applied; the only major difference is the distortion and slight fractures which can be attributed to the applied pressure during demonstration. However, in the reprocessable network, the dye appears more dilute in the matrix after heat and pressure due to the processable nature of the sample, leading to the formation of characteristic flow patterns.

### Classroom Activities

This demonstration was performed in the classroom of a local middle school (7th grade) over two 50 min class periods. A total of ∼80 students participated in a total of 5 classes. During the first class, instructors began with a Kahoot! quiz that asked students questions to gauge their understanding of polymers/plastics followed by a lecture over relevant information to understand the demonstration (all instructional materials and instructor notes can be found in the Supporting Information). The lecture goes over the information in the introduction section. Specifically, we covered a basic introduction of polymers (and their difference from small molecules), the applications of polymers in various subjects, what is recycling and why it is important (which help students understand the importance of sustainability), and common categories of polymers (thermoplastics vs thermosets), as well as reprocessable networks. These topics are crafted to smoothly transition students toward understanding a crucial, albeit slightly complex concept by starting with foundational knowledge, while integrating insights on the challenges and opportunities for enhancing environmental sustainability. Throughout the lecture, we employ examples that can resonate with middle school students, such as the use of plastics for food packaging and textiles, making the scientific principles accessible and facilitating effective learning. This itself is not a part of the demonstration but is necessary to understand the demonstration if lectures on polymer science have not already been given in the existing curriculum. To further aid in student understanding, while the lecture occurred, students were given print-outs of the slides with words removed/blanks to fill in (see Supporting Information). In the second class, the demonstration was introduced, and students were then given vials with the starting materials and asked to make observations guided by a handout. While the networks were prepared by the instructors, students were led through the process with pictures and the formed networks were passed around so they could observe and compare them. The students were asked to make a hypothesis/educated guess about which network would be able to be reformed upon subsequent cutting, heating, and application of pressure. After the demonstration, the students were again asked to write down their observations and evaluate if their hypothesis was correct and explain why using their observations postdemonstration.

The demonstration handout (provided in the Supporting Information) contains some questions to guide the students, which also allow the instructor to assess the learning outcomes. The questions were designed around the learning targets based on Mississippi College- and Career-Readiness Standards for Science^20^ (P.7.5A, P.7.5B (1 and 3), and E.7.9B) where there were four learning targets for the students: (1) define what a polymer is, (2) describe how the two networks are different (based on observation), (3) be able to differentiate recyclable and nonrecyclable materials, and (4) explain how current research is targeting sustainability (Table 1).

**Table 1:** Summary of Pedagogical Goals and How They Were Implemented

Science Standard^a^	Learning Target	Implementation
P.7.5A: Students will demonstrate an understanding of the physical and chemical properties of matter.	(1) Define what a polymer is and (2) describe how the two networks are different	Class 1: Kahoot!, lecture, and exit ticket
		Class 2: Observation, demonstration, and handout
P.7.5B: Students will demonstrate an understanding about the effects of temperature and pressure on physical state, molecular motion, and molecular interactions.	(3) Be able to differentiate recyclable and nonrecyclable materials	Class 1: Kahoot! and lecture
		Class 2: Observation and demonstration
E.7.9A: Students will demonstrate an understanding of the relationship between natural phenomena, human activity, and global climate change.	(4) Explain how current research is targeting sustainability	Class 1: Kahoot! and lecture
		Class 2: Demonstration

a From 2018 Mississippi College- and Career-Readiness Standards for Science for 7th graders.

The design of the lesson implemented several pedagogical strategies including active learning and game-based learning. Previous educational literature has demonstrated that active learning increases student engagement and performance.^21−23^ Since active learning is widely integrated into the K–12 classroom, it was strategically integrated into the presentation of this lesson. Specifically, game-based learning^24^ and multimedia learning were implemented through the Kahoot! quiz since the students used the class set of laptops to complete the quiz. Think-pair-share^25^ was also incorporated; when a question was asked, students first developed their own thinking prior to discussing their responses with classmates and subsequently did their worksheets in groups. The design of hands-on experiments and demonstrations to help students visualize the difference in processability in distinct samples leverages the experiential learning strategy.^26^ Bloom’s taxonomy^27^ was also employed during the planning; first, the focus was on simply communicating the information, and by the end of Day 2, higher-level thinking was required to complete the worksheet with peer discussion encouraged.

While this demonstration/lesson plan was originally designed for seventh grade science students in Mississippi, the learning standards state to state in the U.S. are similar; therefore, adapting this to fit individual classrooms should be relatively simple with the instructor notes provided. Potential room for improvement is also identified in the Supporting Information along with suggested solutions. For example, if your classroom does not have the capability for every student to have a phone/laptop to play the Kahoot!, we recommend adapting the questions into another type of game, so it is still a fun introduction for the students. Opportunities also exist for the application of this demonstration in a high school or even college setting as a hands-on experiment. Details are in the Supporting Information, but this is proposed to take place over four class periods with day 1 as lecture, day 2 for preparing the two samples, day 3 for curing the samples, and day 4 for testing reprocessability.

### Students’ Response

The students were quite excited for the Kahoot! quiz, and starting with the use of multimedia (e.g., laptops) helped to grab their attention for the rest of the lecture. They were engaged throughout the lessons, particularly during observation of the demonstrations. Students were also excited when they were given gloves and interacted with different polymer networks, all derived from common household foods. Overall, the students had a fun time with the hands-on, nontraditional lesson, and the chosen pedagogical approaches were found to be successfully implemented.

### Test Lesson

Handouts were given at the conclusion of each lecture to determine middle school students’ level of understanding and evaluate the learning objectives. This demonstration was performed for ∼80 students over a two-day period. After day one, the students were given an exit ticket and asked to write down a new definition that they learned and what they wanted to learn more about on the second day. This was done to see if they would be able to give the definition of a polymer or polymer related term. When reviewing their answers, it was found that 56% did not leave with a good understanding and/or could not communicate clearly; 31% understood and were able to communicate clearly; and 13% excelled and exceeded expectations. These categories were based on their responses to the question of what they wanted to learn about in the next meeting (building on what was covered during this class) and were made by comparing their answers to each other’s. More specifically, among the 56% who did not leave with a good understanding and/or could not communicate clearly, more than 50% of them seemed to have an understanding about overarching concepts (such as describing the reprocessing and cross-linking nature), but their use of scientific terminology was incorrect (i.e., switched up the words “monomer” and “polymer”). Answers varied from “polymers” to complex concepts regarding some of the current research that is being done in universities that was briefly mentioned during the lecture.

After the second day, the handout asked questions probing students’ understanding of the demonstration. We note that the question sheet (for learning outcome assessment) as well as the answer key are included in the Supporting Information, which teachers can either directly use or tailor to address their own class. Through our demonstration, it is found that 84% of students can effectively describe what happened to the materials during curing (from Concluding Question 1), 58% of students recognized the need of adding baking soda as a catalyst (from Concluding Question 2), and 62% of students were able to understand the concept of reprocessing networks and their difference compared to permanently cross-linked networks (from Concluding Questions 3–5). Concluding Question 6 is an open question, in which 85% of students provided an answer that explained how the results confirmed or refuted their original hypotheses. We believe these results confirm that our demonstration was grade-appropriate and successfully indicated the students’ understanding of the learning targets. Therefore, this demonstration could effectively help middle school students to grow their knowledge in polymer materials as well as be further aware of sustainability needs.

## Summary

This work presents a middle school demonstration aiming to increase the general knowledge of sustainable polymers along with showing how polymer scientists are currently developing new materials to address the plastic waste problem. The materials used in this demonstration include epoxidized soybean oil, citric acid, and baking soda, all common household, bioderived food items that are readily available in grocery stores. The procedures described do not involve high-cost equipment and are suitable for middle school students. In the Supporting Information, we provide all PowerPoint presentations and handouts used as well as thorough instructor notes. The students are asked thought provoking questions to assess their understanding of polymers/plastics and are brought through the process of making the networks, and the reprocessability of the dynamic networks is demonstrated in real-time.

## Supporting Information Available

Lecture Slides (PDF)
Reprocessable Networks Handout; Conclusion Questions; Key to Answers (PDF)
Instructor Notes (PDF)
