# Supplementary material for: Reprocessable Networks from Vegetable Oils, Salts, and Food Acids: A Green Polymer Outreach Demonstration for Middle School Students
Source: J Chem Educ. 2024 Jun 5;101(7):2947–53. doi: 10.1021/acs.jchemed.3c01258 (PMC11238532; doi:10.1021/acs.jchemed.3c01258)

Supporting Information for:

Reprocessable Networks from Vegetable Oils, Salts, and Food Acids: A Green Polymer Outreach Demonstration for Middle School Students

Sara Valdez[1], Carmen Dunn[1], Miya Hullum[2], Evains Harper[3], and Zhe Qiang[*,1]

[1] School of Polymer Science and Engineering, The University of Southern Mississippi 118 College Drive, Hattiesburg, MS 39406

[2] Hattiesburg High School 301 North Hutchinson Avenue, Hattiesburg, MS 39401

[3] N.R. Burger Middle School 174 W.S.F. Tatum Blvd, Hattiesburg, MS 39401

* Corresponding Author (Email: zhe.qiang@usm.edu)

Class 1

Lecture on polymers and reprocessable networks

Kahoot!

What is a polymer???

Polymers are large molecules made up of many repeating units!

Each repeating unit is called a **monomer**!

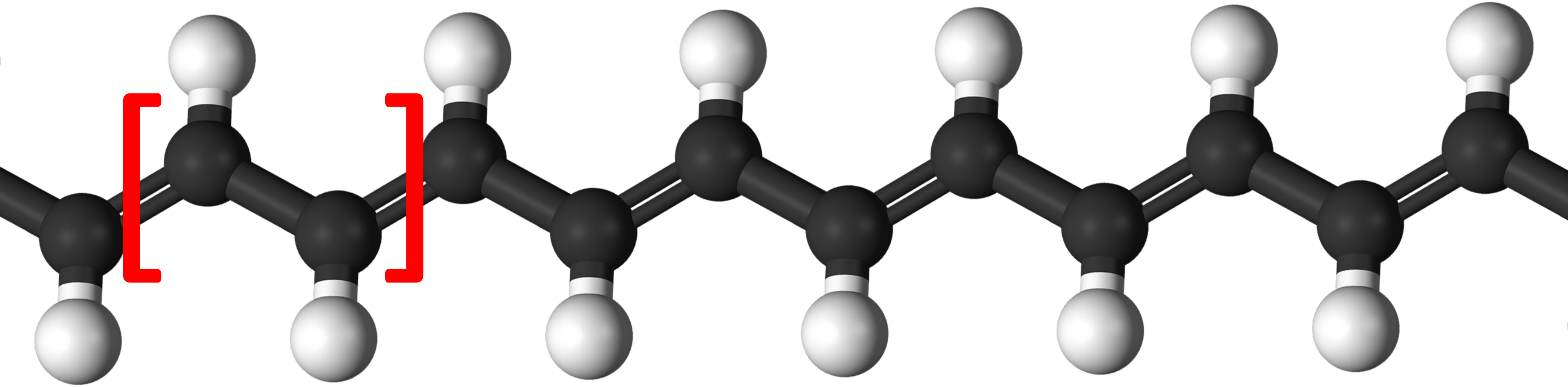

poly = many
mono = one
mer = units

How many atoms are in each "monomer" unit?

Polymers are all around us!

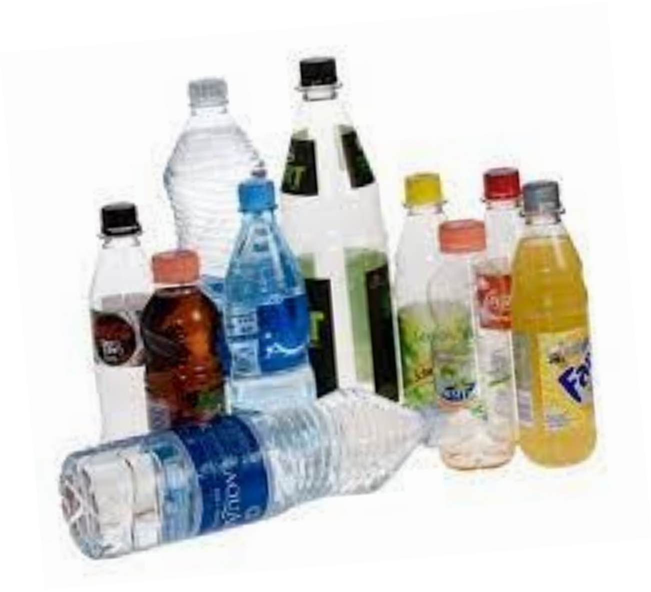

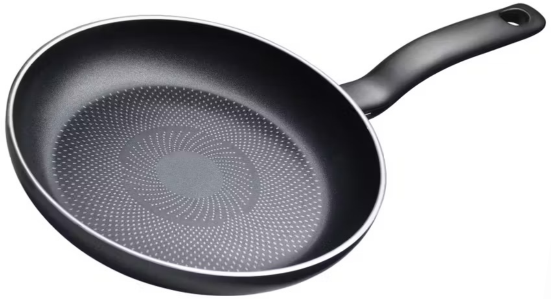

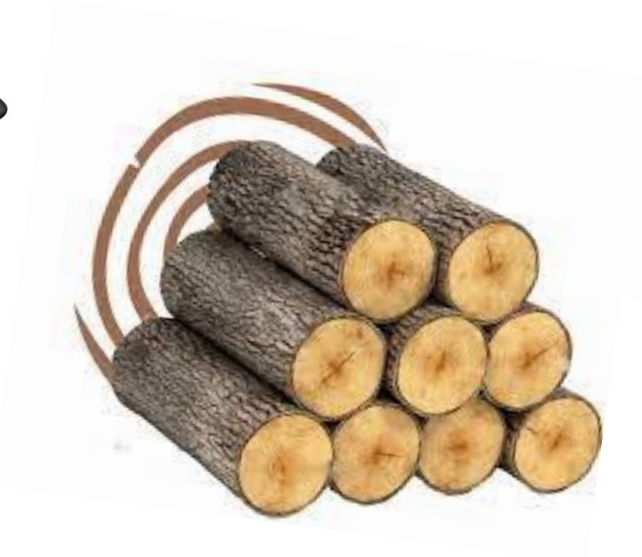

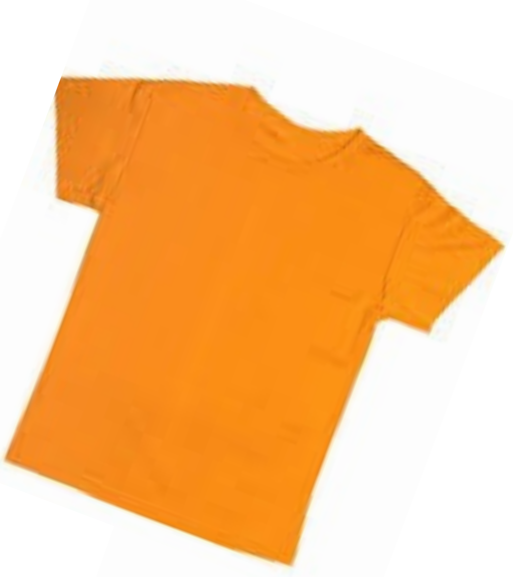

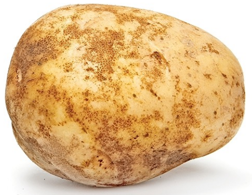

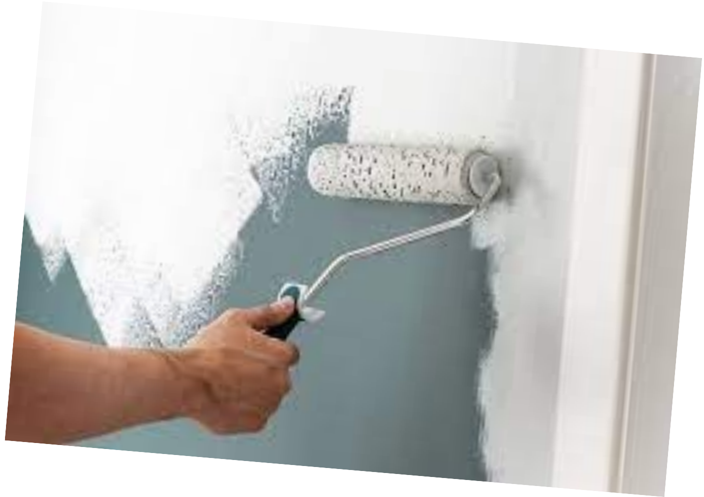

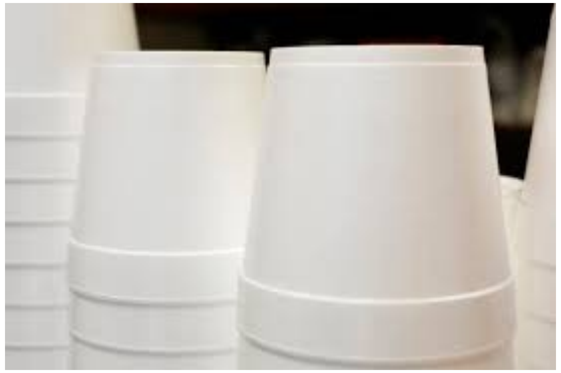

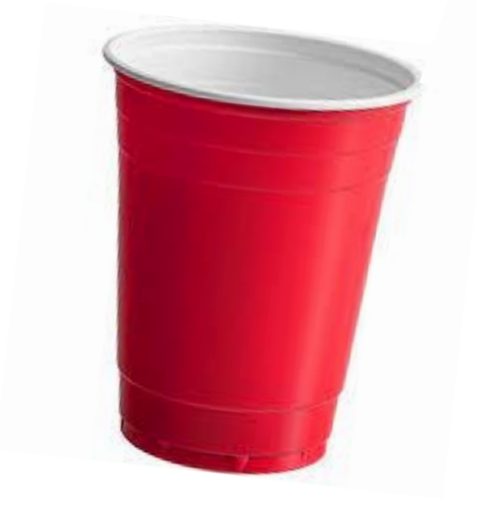

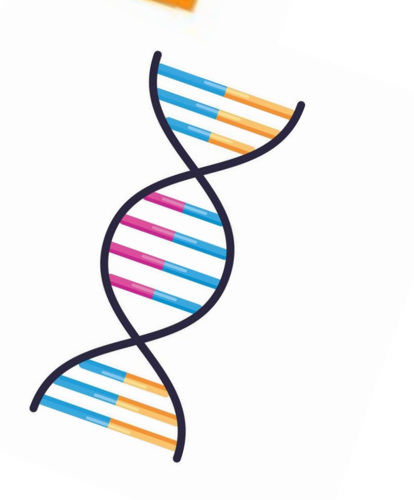

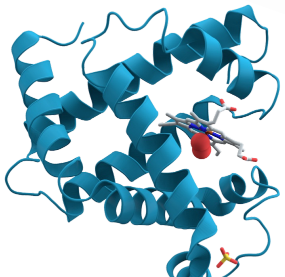

What is recycling, and why is it important?

Definition: recycling is converting waste polymers into reusable materials

Recycling lowers the amount of waste in landfills, decreases pollution, and conserves resources.

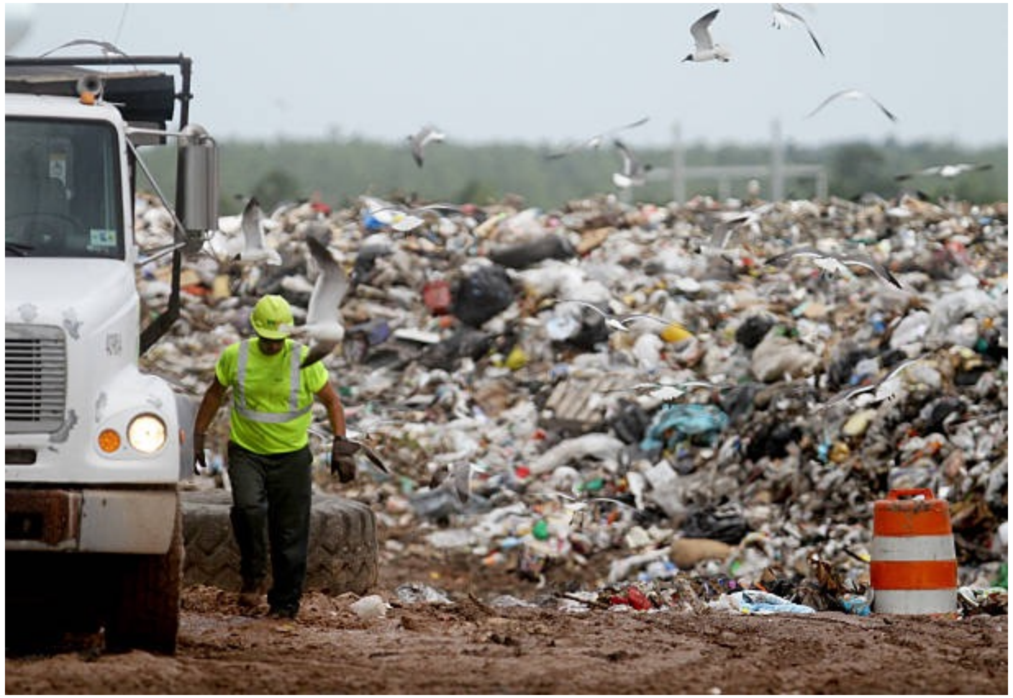

- **3,579,091 tons** of waste was sent to Mississippi landfills in 2019 alone.
- That means each of the **2,978,000 residents** of Mississippi in 2019 produced about **1.2 tons** of waste!

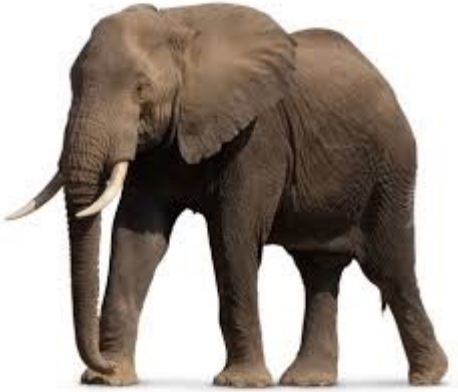

That's the weight of a small elephant!

Can we recycle every plastic?

NO!

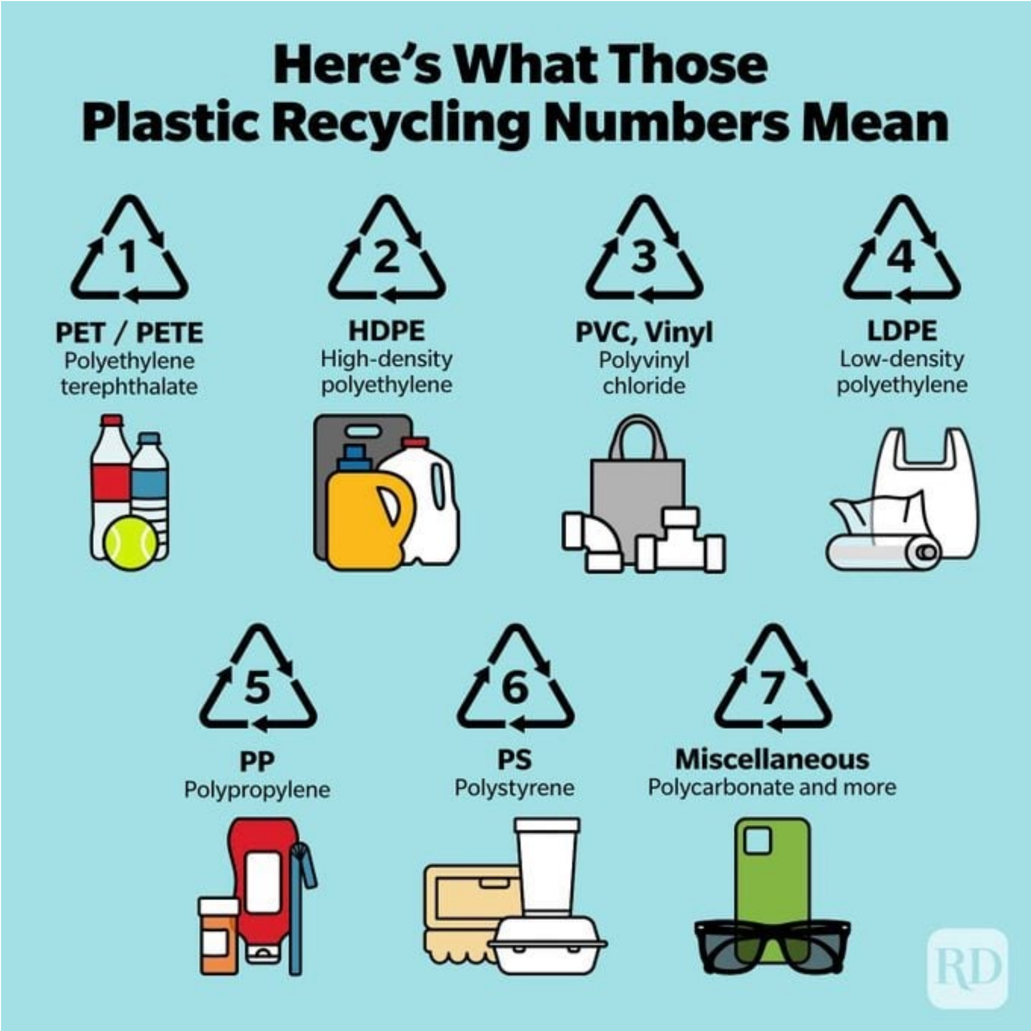

There are two main types of polymers!

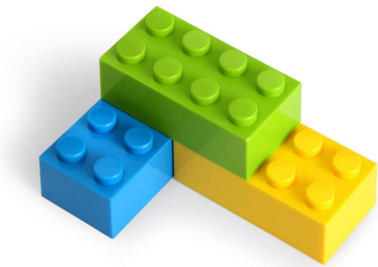

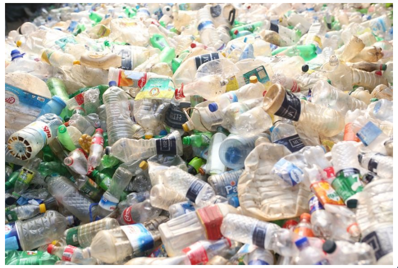

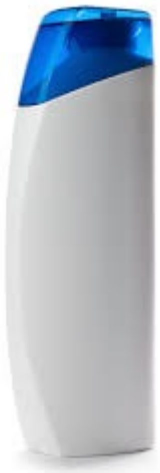

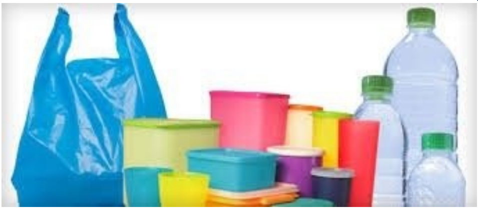

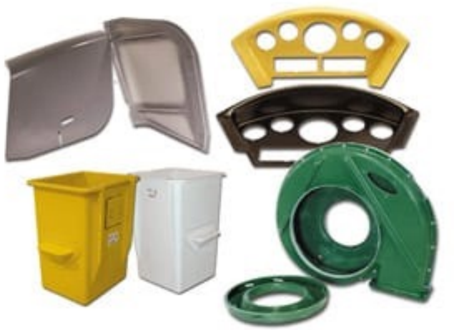

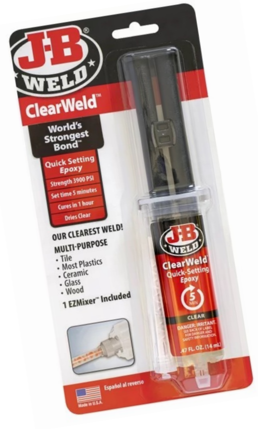

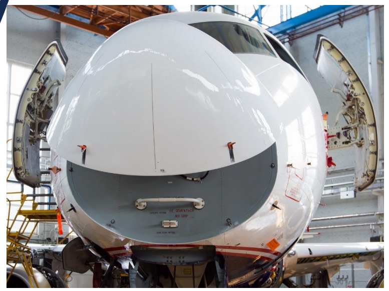

Thermoplastics

- Can melt when heated
- **Can be recycled**
- Better properties at lower temperatures
- More expensive
- Molecules are long **linear** chains

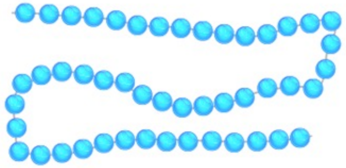

?

Thermosets

- Do not melt when heated
- **Cannot be recycled**
- Cheaper materials
- Good for high temperatures
- Molecules are "**networks**" of connected chains
- Connections are called "**crosslinks**"

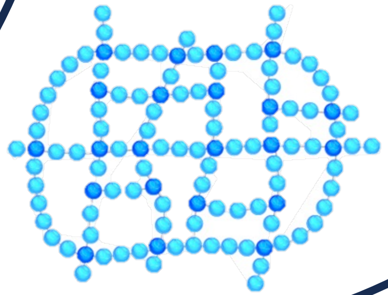

What is a reprocessable network?

Definition: A reprocessable network is a thermoset with crosslinks that can be formed and broken.

This lets us make "thermosets" that can be melted and potentially recycled!

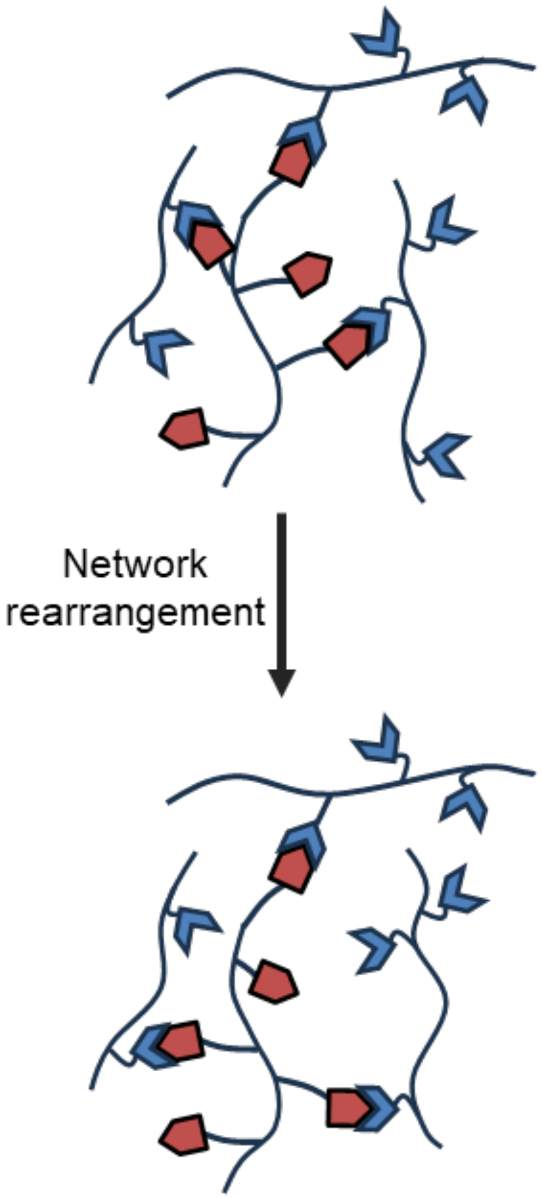

Extension

This space was used to discuss current research in polymer science sustainability over the course of 5 slides.

What questions do you have?

Exit Ticket!

1. Write down one definition we talked about today.

2. What is one thing you are interested in learning more about?

Class 2

Review of polymers and reprocessable networks

Demonstration

Review from Introduction to Polymers

What is a polymer?

Thermoset example?

Thermoplastic example?

Anything else you remember?

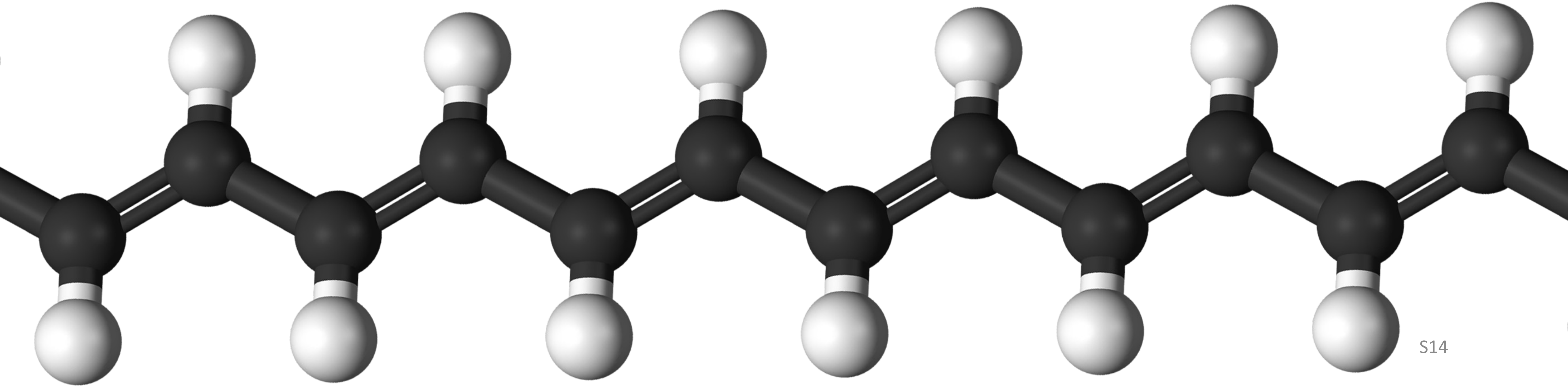

There are two main types of polymers!

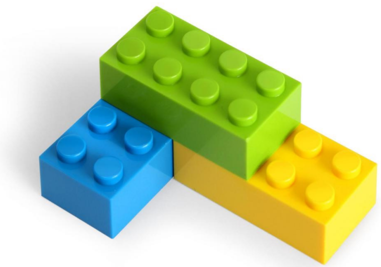

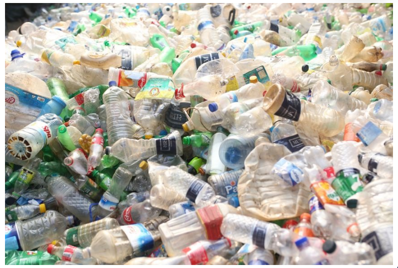

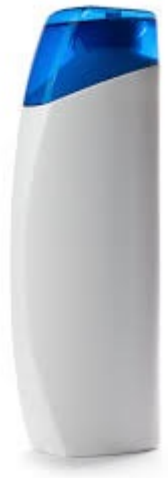

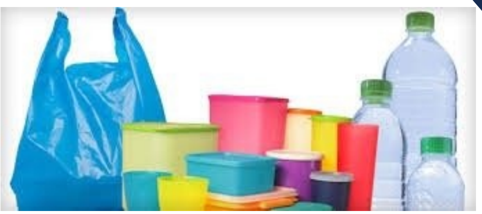

Thermoplastics

- Can melt when heated
- **Can be recycled**
- Better properties at lower temperatures
- More expensive
- Molecules are long **linear** chains

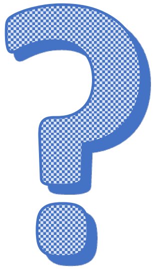

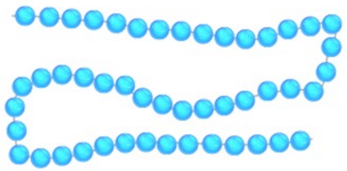

Thermosets

- Do not melt when heated
- **Cannot be recycled**
- Cheaper materials
- Good for high temperatures
- Molecules are "**networks**" of connected chains
- Connections are called "**crosslinks**"

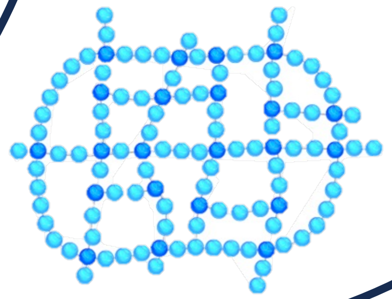

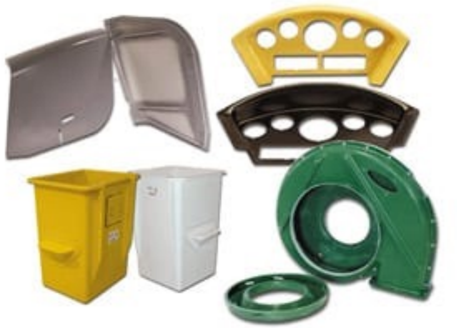

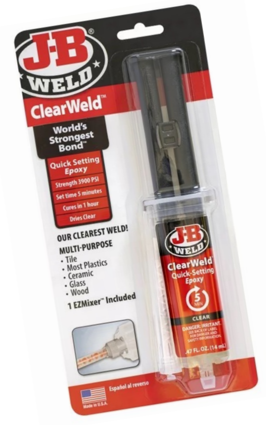

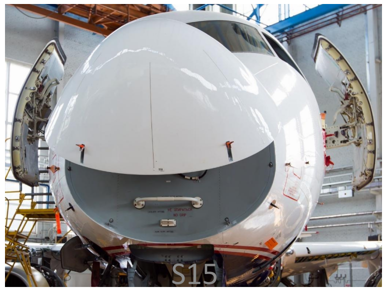

What is a reprocessable network?

Definition: A reprocessable network is a thermoset with crosslinks that can be formed and broken.

This lets us make "thermosets" that can be melted and potentially recycled!

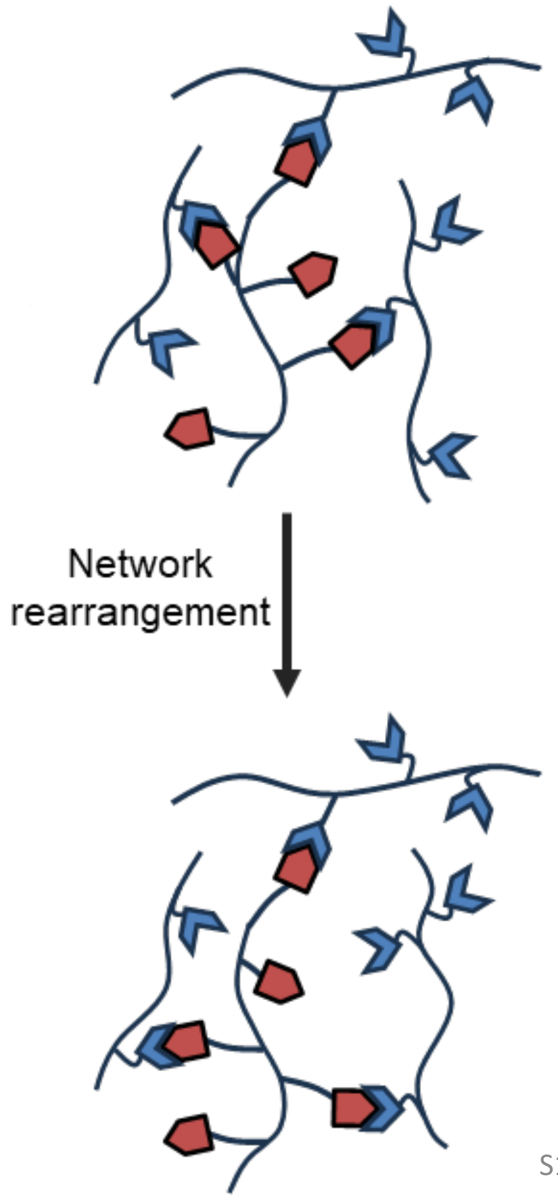

What is a chemical reaction and how do we tell if one has occured?

Chemical reaction: a process that leads to the chemical transformation of one material to another

Color change

Forms a solid

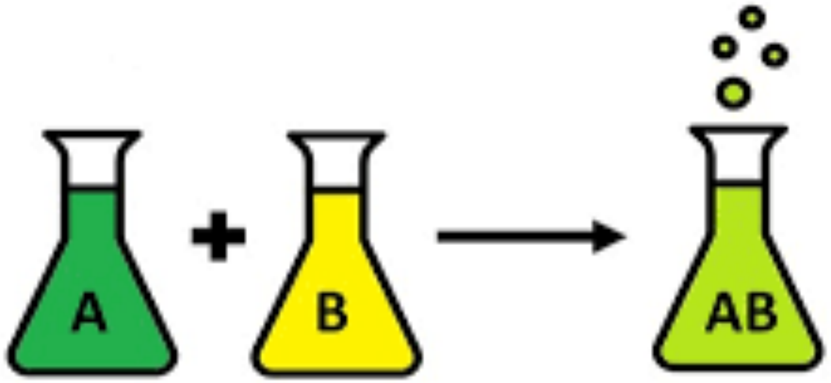

Bubbling/gas formation

Produces a smell

Change in temperature

What causes chemical reactions?

Functional group: a specific arrangement of atoms that allows a reaction to happen

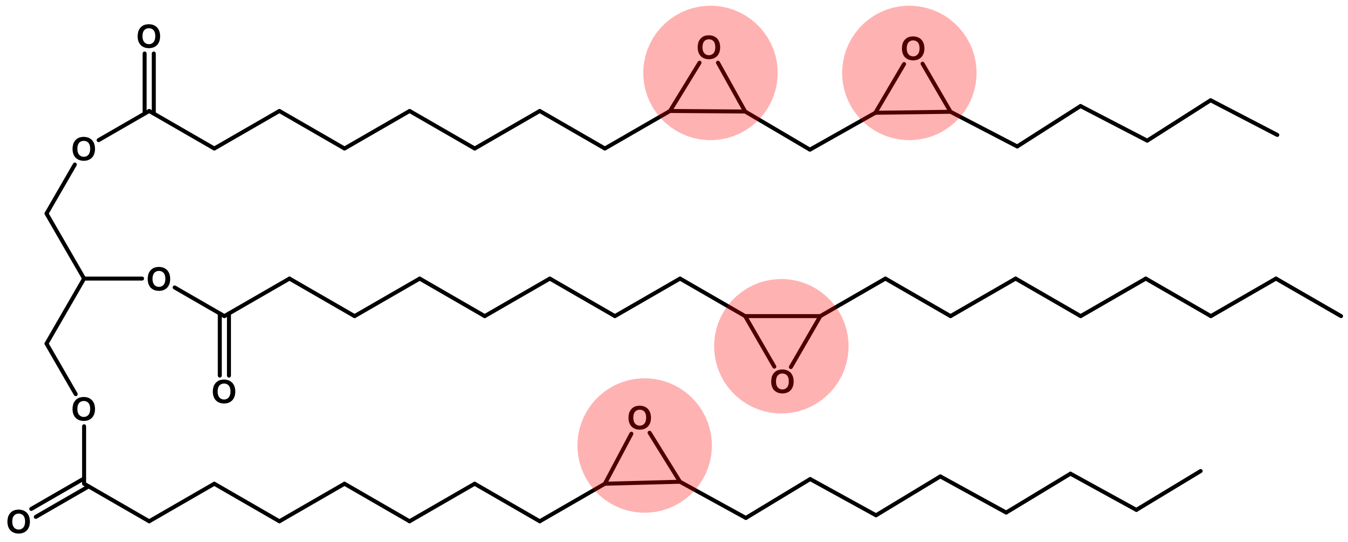

Epoxidized Soybean Oil (ESO)

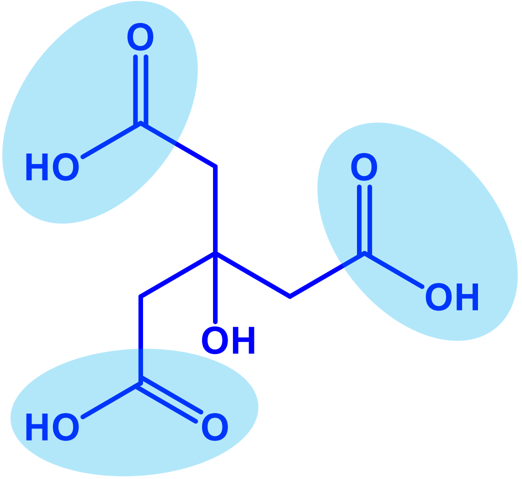

Citric Acid

Catalyst: a substance that makes a reaction go faster or more easily (example: less heat needed)

Reprocessablity Demonstration!

Thermoset

- Soybean oil
- Citric acid (crosslinker)
- Ethanol (solvent)

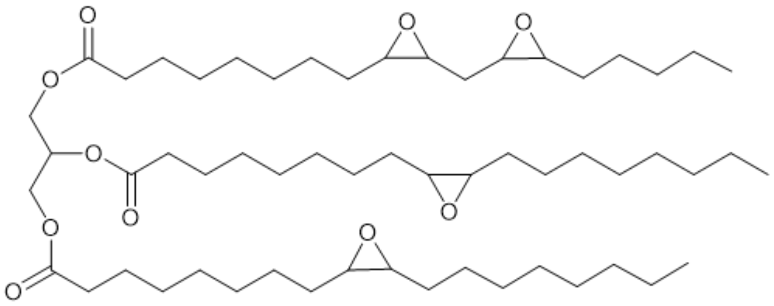

Epoxidized soybean oil (ESO)

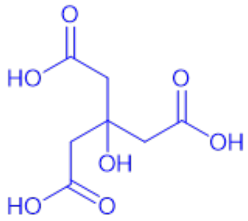

Citric acid

Reprocessable Network

- Soybean oil
- Citric acid (crosslinker)
- Baking soda (catalyst)
- Ethanol (solvent)

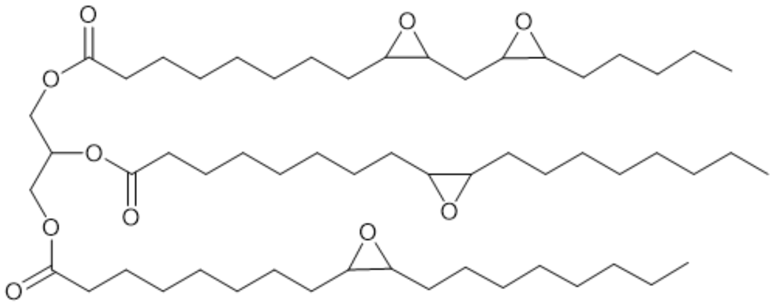

Epoxidized soybean oil (ESO)

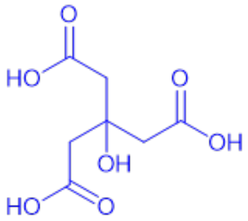

Citric acid

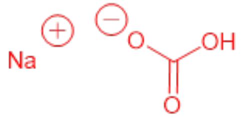

Sodium bicarbonate

What do the starting materials look like?

Let's take ~5 minutes to write our observations on the handout!

Citric acid
in ethanol

Baking
soda in
water

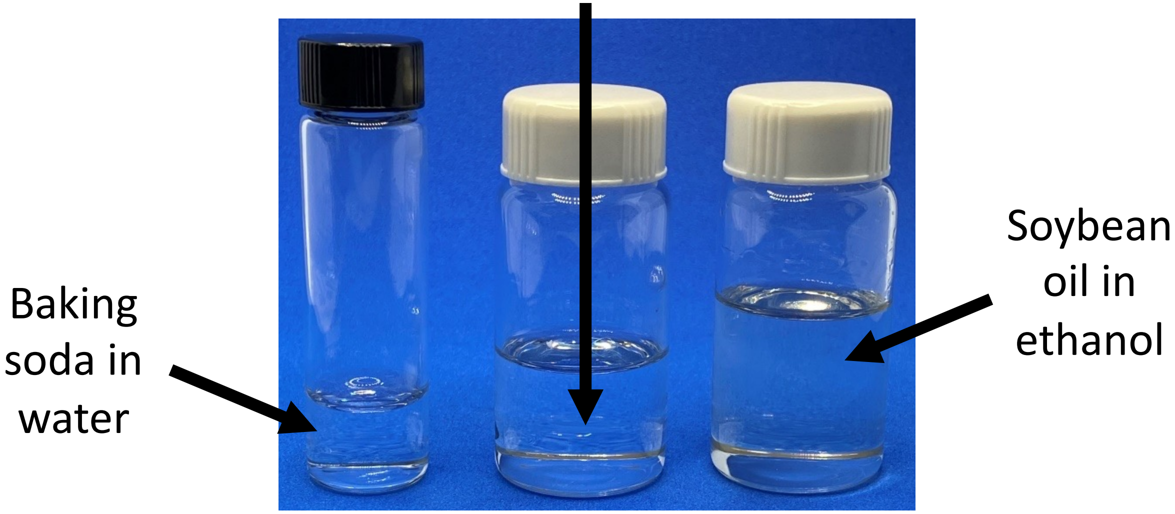

Soybean
oil in
ethanol

How the samples are made:

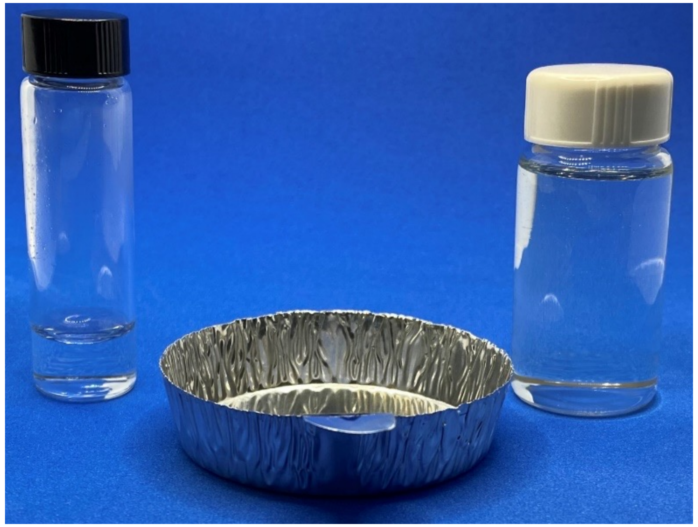

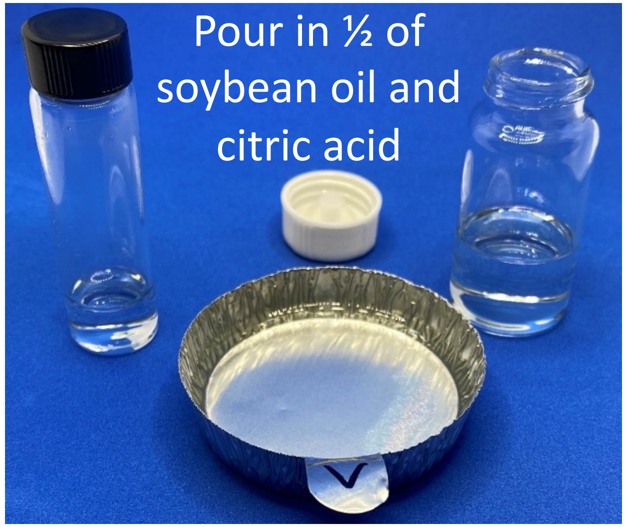

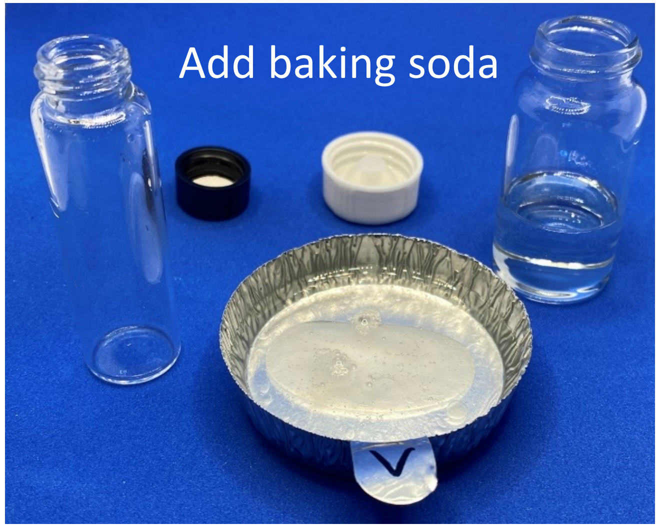

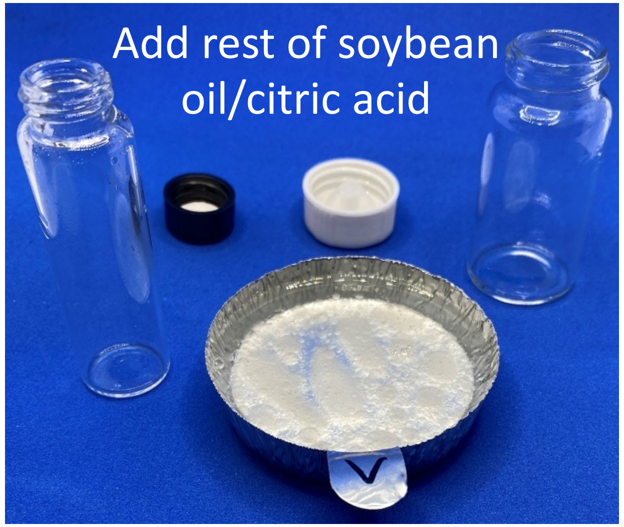

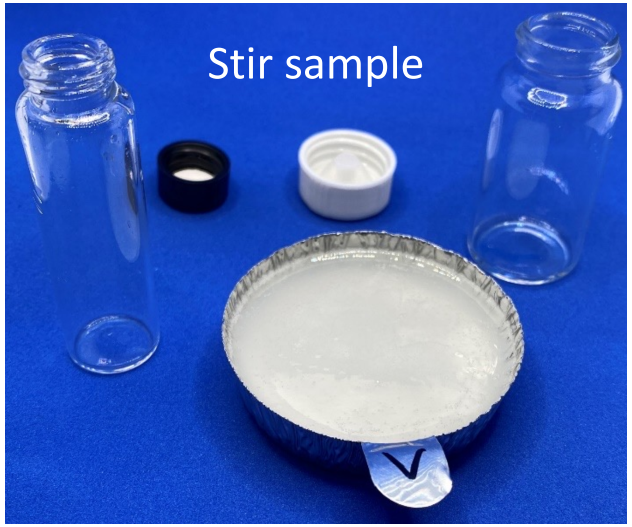

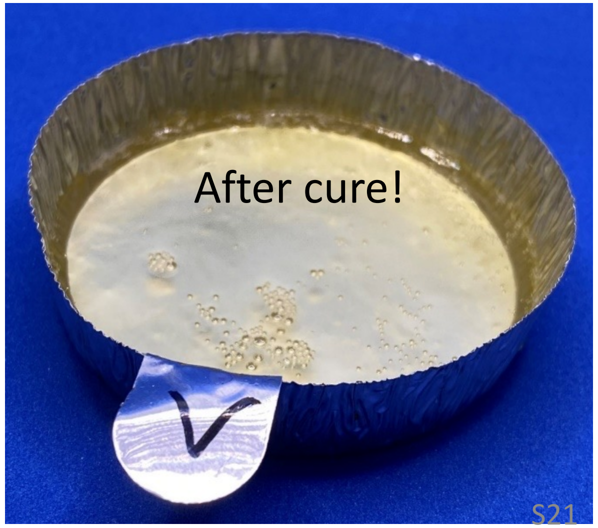

What do the cured samples look like?

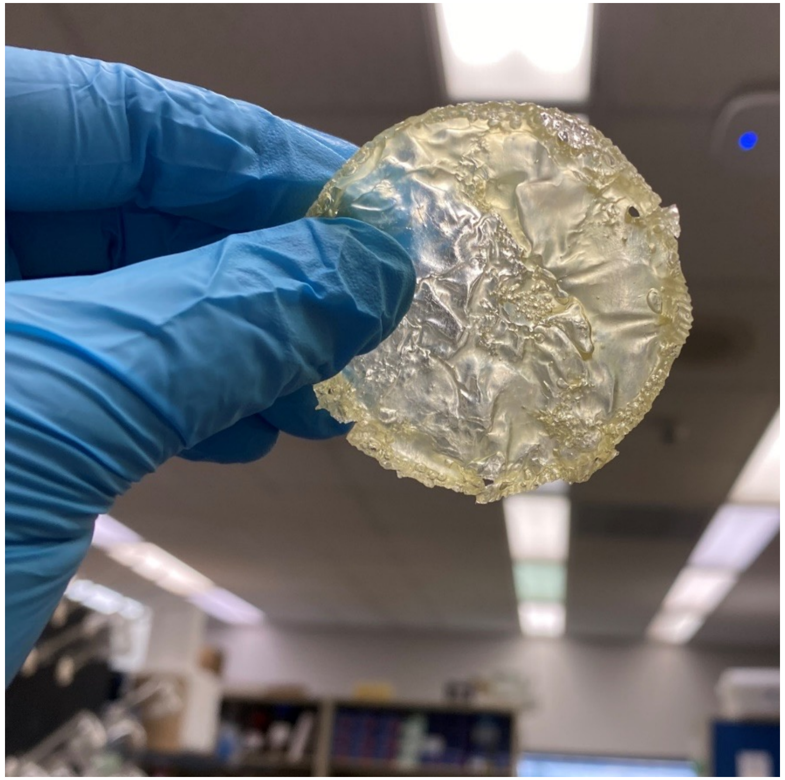

After cure, they are mostly clear, with a yellowish tint. They are sticky but solid and can be peeled out of the aluminum pan.

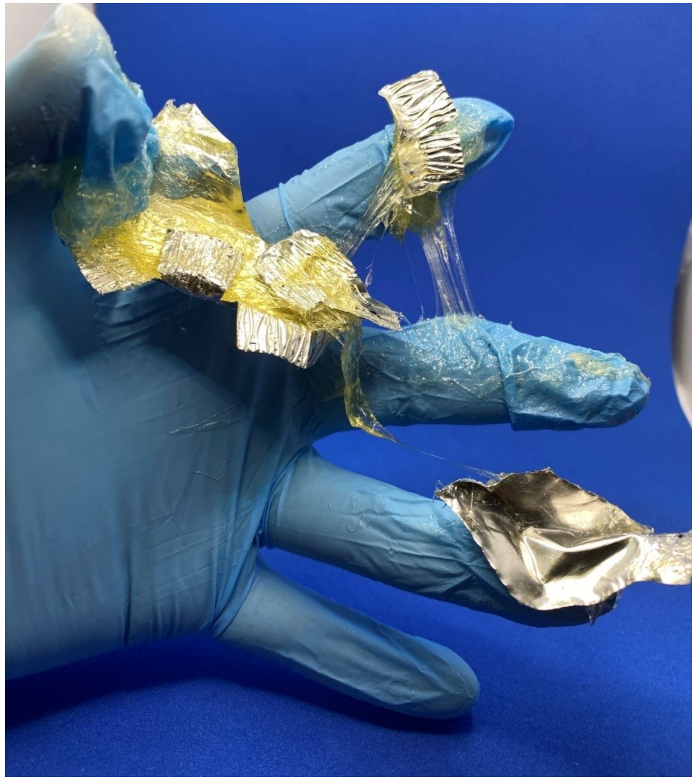

If they don't fully react, they are extremely sticky, gooey, and stretchy! These cannot be taken out of the pan.

Let’s do the reprocessing!

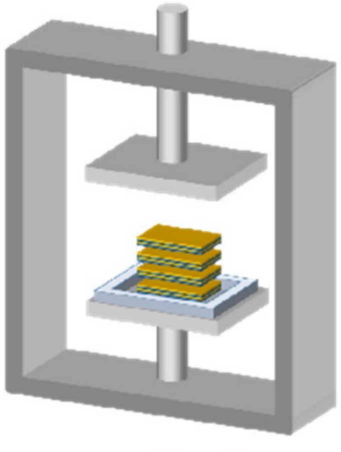

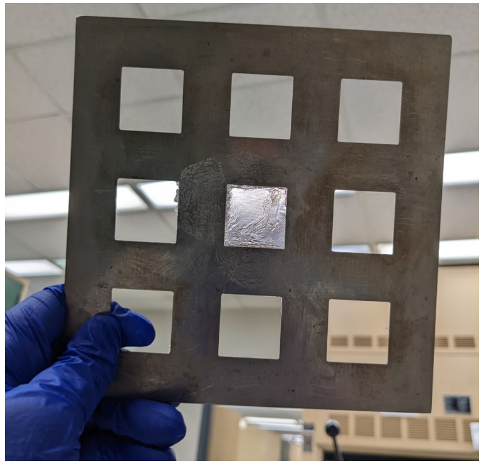

We are mimicking melt pressing. In melt pressing, the sample is stacked up and sandwiched between two hot metal plates and pressure is added to “squish” the sample into a new shape.

Procedure

1. Cut sample into two pieces
2. Lay one piece in the glass petri dish
3. Lay the other piece on top of the other so that is overlapping
4. Add the top of the petri dish
5. Put the petri dish on the hot plate (set to 125 °C)
6. Add the weight to the top of the petri dish
7. Wait ~5-10 minutes

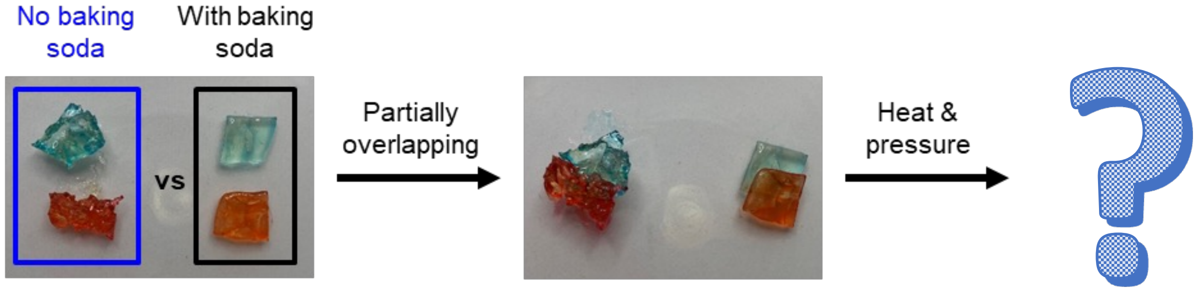

Write your hypothesis on the handout!

Was your hypothesis correct?

Make sure to fill out the second sheet of the handout before class is over!

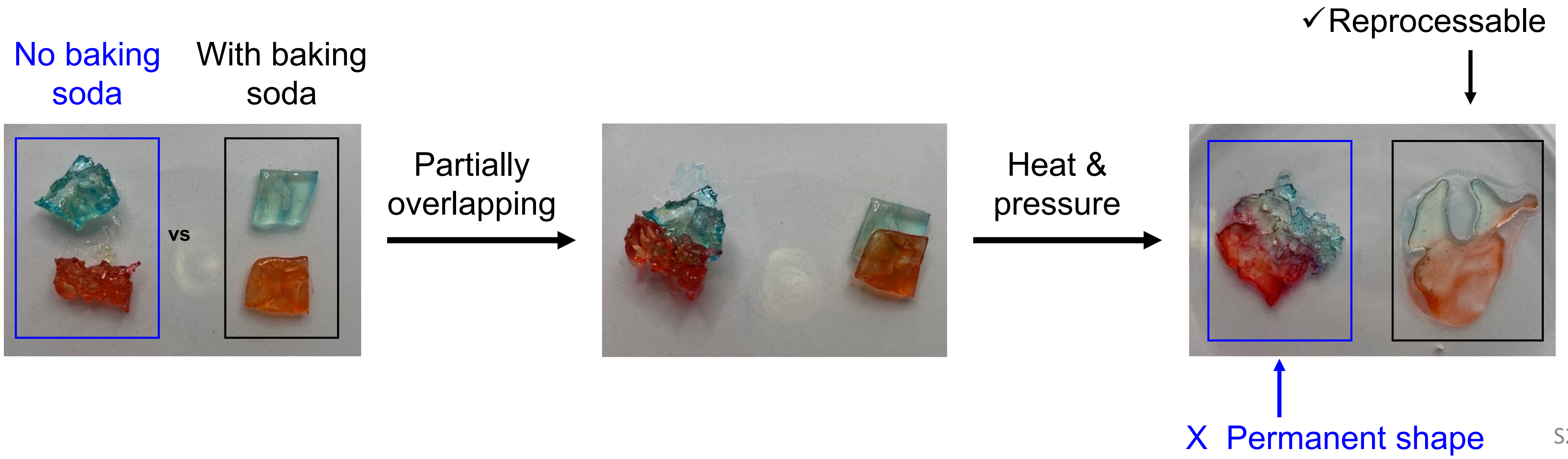

Supplement: Supplementary file 1 — ed3c01258_si_001.pdf [file ed3c01258_si_001.pdf]
