# Supplementary material for: Reprocessable Networks from Vegetable Oils, Salts, and Food Acids: A Green Polymer Outreach Demonstration for Middle School Students
Source: J Chem Educ. 2024 Jun 5;101(7):2947–53. doi: 10.1021/acs.jchemed.3c01258 (PMC11238532; doi:10.1021/acs.jchemed.3c01258)
Supplement: Supplementary file 2 — ed3c01258_si_002.pdf [file ed3c01258_si_002.pdf]

Supporting Information for:

Reprocessable Networks from Vegetable Oils, Salts, and Food Acids: A Green Polymer Outreach Demonstration for Middle School Students

Sara Valdez[1], Carmen Dunn[1], Miya Hullum[2], Evains Harper[3], and Zhe Qiang[*,1]

[1] School of Polymer Science and Engineering, The University of Southern Mississippi 118 College Drive, Hattiesburg, MS 39406

[2] Hattiesburg High School 301 North Hutchinson Avenue, Hattiesburg, MS 39401

[3] N.R. Burger Middle School 174 W.S.F. Tatum Blvd, Hattiesburg, MS 39401

* Corresponding Author (Email: zhe.qiang@usm.edu)

Name: ____________________ **Class Period:** ______

Question: what is a reprocessable network?

Describe our starting materials!

Epoxidized Soybean Oil (ESO):

Color:

State of matter:

Other descriptors:

Citric Acid:

Color:

State of matter:

Other descriptors:

Ethanol:

Color:

State of matter:

Other descriptors:

Sodium Bicarbonate (Baking Soda):

Color:

State of matter:

Other descriptors:

Hypothesis: what do you think the baking soda will do to this reaction?

Name: ______________________ **Class Period:** _______

Concluding Questions: what did we learn?

1. **What happened when the materials were cured?**

2. **What was the purpose of adding baking soda?**

3. **What happened to the thermoset when we applied heat and pressure?**

4. **What happened to the reprocessable network when we applied heat and pressure?**

5. **What differences do you notice between the materials?**

6. **Did your observations support your hypothesis? Why or why not?**

Name: ANSWER KEY ______________ **Class Period:** _______

Question: what is a reprocessable network?

A reprocessable network is a thermoset with crosslinks that can be formed and broken.

Describe our starting materials!

Epoxidized Soybean Oil (ESO):

Color: colorless/clear

State of matter: liquid

Other descriptors: Anything pertaining to viscosity, comparisons drawn to other liquids, "greasy", "looks like _"

Citric Acid:

Color: white

State of matter: solid

Other descriptors: Anything pertaining to size of crystal/powder, or general appearance, "looks like _"

Ethanol:

Color: colorless/clear

State of matter: liquid

Other descriptors: Anything pertaining to appearance, how the ethanol refracts light, "looks like _"

Sodium Bicarbonate (Baking Soda):

Color: white

State of matter: solid

Other descriptors: Anything pertaining to size of crystal/powder, or general appearance, "looks like _"

Hypothesis: what do you think the baking soda will do to this reaction?

My hypothesis is _______.

When heat and pressure are applied to the reprocessable network, _____ will happen.

When heat and pressure are applied to the thermoset, _____ will happen.

Name: ANSWER KEY Class Period: _______

Concluding Questions: what did we learn?

1. **What happened when the materials were cured?**

The materials became solid networks when cured.

2. **What was the purpose of adding baking soda?**

The baking soda was a catalyst to allow the reprocessable network to continue reacting with heat and pressure.

3. **What happened to the thermoset when we applied heat and pressure?**

When heat and pressure were applied to the two pieces of the thermoset, they remained as two separate pieces (no change).

4. **What happened to the reprocessable network when we applied heat and pressure?**

When heat and pressure were applied to the two pieces of the reprocessable network, they joined together to form one reconnected network or piece.

5. **What differences do you notice between the materials?**

Students can use this space to talk about how the phenomena resembled melting or the lack of reaction in the thermoset or talk about where reactions happened or did not happen.

6. **Did your observations support your hypothesis? Why or why not?**

This is an open answer question for students to compare their observations to their original hypothesis with explanation.
