# Supplementary material for: Reprocessable Networks from Vegetable Oils, Salts, and Food Acids: A Green Polymer Outreach Demonstration for Middle School Students
Source: J Chem Educ. 2024 Jun 5;101(7):2947–53. doi: 10.1021/acs.jchemed.3c01258 (PMC11238532; doi:10.1021/acs.jchemed.3c01258)
Supplement: Supplementary file 3 — ed3c01258_si_003.pdf [file ed3c01258_si_003.pdf]

Supporting Information for:

Reprocessable Networks from Vegetable Oils, Salts, and Food Acids: A Green Polymer Outreach Demonstration for Middle School Students

Sara Valdez[1], Carmen Dunn[1], Miya Hullum[2], Evains Harper[3], and Zhe Qiang[*,1]

[1] School of Polymer Science and Engineering, The University of Southern Mississippi 118 College Drive, Hattiesburg, MS 39406

[2] Hattiesburg High School 301 North Hutchinson Avenue, Hattiesburg, MS 39401

[3] N.R. Burger Middle School 174 W.S.F. Tatum Blvd, Hattiesburg, MS 39401

* Corresponding Author (Email: zhe.qiang@usm.edu)

Instructor Notes

BACKGROUND

Prior to this demonstration, students should have some understanding of states of matter, the scientific method, temperature/pressure relationships, and have been introduced to atoms/the periodic table. The completion of this demonstration allows students a better understanding of the relationship between heat, pressure, and processability of polymer networks as well as introduce sustainability as a socioscientific issue to help inspire future generations to attack the plastic waste problem.

Melt pressing is frequently used in the polymer industry as a processing method for achieving the appropriate size and shape of a plastic part with desired properties. However, this technique is only applicable to processable (recyclable) materials and typically excludes thermosetting networks. Alternatively, the creation of a dynamic or reprocessable network allows for moldable materials to be made with different properties; this means networks could potentially be recycled. This lesson defines thermosets and thermoplastics as the two main types of polymers and introduces reprocessable networks for students with fundamental knowledge of chemical reactions. Completion of this lesson also connects concepts of climate change, sustainability, plastics recycling, and the scientific method. All materials made for demonstration purposes originate from readily available household 'chemicals' and illustrate the applications of polymer chemistry to everyday life.

This activity presents two classes of content to introduce polymer terminology and demonstrate the creation of new materials to potentially address recycling challenges, all achievable in a classroom setting. Originally applied to a 7th grade science classroom in Mississippi, this activity fulfilled several state standards, demonstrating heat/pressure relationships for polymer systems, the scientific method, and climate change.

This demonstration is applicable for regular instruction in 6th-8th science classrooms, but the opportunity to extend this as an experiment in a high school setting exists. More information on the adaptation for high school students can be found in **Additional Information** at the end of this document.

CLASS 1 (~50-minute class period)

Prior to beginning this activity, it is recommended to teach a lesson on general polymer science terminology and introduce thermoplastics, thermosets, and reprocessable networks. In this case, this lesson/demonstration would take approximately two class periods (55 minutes each). For 7th-12th grade state curriculums, polymer science is typically mentioned but rarely in detail.

It is recommended to introduce terminology through presentation and classroom games to help grab students' attention. The authors used a Kahoot! quiz (Link to Kahoot!: https://create.kahoot.it/share/polymer-science/095d1ea1-f3c7-4765-b56f-b89a5f047846) to introduction some of the main points in the lecture and then taught a lesson with a PowerPoint presentation. The link to the Kahoot! can also be found in the notes on the Kahoot! slide in **Lecture Slides (PPTX)**. In this class period, the instructors also presented modern research on reprocessable networks and polymer sustainability taking place at their university. We also recommend researching statistics about recycling/landfill management in your local area. We

found students connected to these numbers and asked more engaging questions during this section of the presentation. At the close of this class period, students completed an exit ticket where they wrote down one definition they learned and one concept they were interested in learning more about. From these responses, the instructor can gauge student understanding and clarify concepts at the beginning of the second class.

By the end of this first lesson, students should be able to:

- Define 'polymer' and 'monomer'
- Differentiate between thermoplastics and thermosets
- Explain how reprocessable networks address recycling challenges

CLASS 2 (~50-minute class period)

The first few minutes of the second class should be used to clarify any misconceptions or questions from the end of the first lecture. As described in the main text, vials of starting materials are passed around the class for observation. It is advised to have the starting materials prepared for students before class; they will record their observations on the **Reprocessable Network Handout**. During the introduction on this class, begin by asking the question of "what is a reprocessable network" (other probing questions are on the slides as well—ensure they write down their answers on the handout). Ask students to contribute to the discussion and recall any information they can from the last lesson.

We recommend connecting concepts of chemical reactions to these materials. Have students recall the states of matter, what indicates a chemical reaction, and any other descriptors that could be used to indicate the occurrence of a chemical reaction. Then, review the scientific method and define functional groups. For the middle school classroom, the idea of functional groups will be entirely foreign (as it is not introduced until college level organic chemistry). So, the authors introduced functional groups as being specific arrangements of atoms that are the points that chemical reactions take place at on a molecule. Any mechanisms will also be beyond their level at this point, the dynamic nature of the reprocessable network is illustrated with graphics in the lecture slides.

Next, introduce that you will be making thermosets and reprocessable networks by reacting epoxidized soybean oil (ESO) with citric acid. First, the students need to look at the starting materials. Pass out boxes containing the starting materials (ESO, citric acid, ethanol, and baking soda) and have students write down observations of each material (Figure S1). From experience, student discussion in groups of 2-3 works best and encourages more in-depth thought (and requires less preparation of the starting materials). It is recommended to give the students ~5 minutes to complete their observations. After the students are done with their observations, give the students an additional 2 minutes to work in groups to form their hypothesis on what will happen to the two networks as they are exposed to heat and pressure.

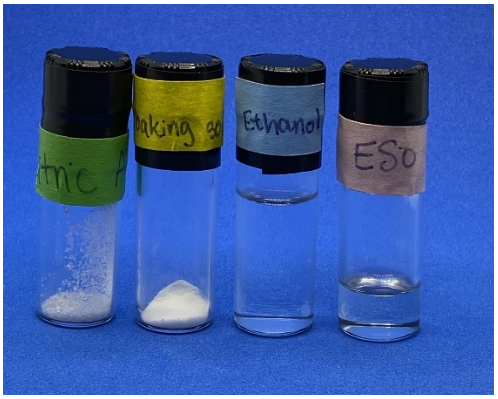

Figure S1. Starting materials passed around to students for initial observation with different colored labels, from left to right: citric acid, baking soda (sodium bicarbonate), ethanol, and epoxidized soybean oil (ESO).

Then the students will be led through the process of making the two networks. When this demonstration was performed, all of the samples were made ahead of time in the lab and the students were shown pictures of the entire process. However, if there is enough time, the two networks can be made in front of the students as part of the demonstration. If this is done, it is recommended to have all the materials preweighed and another sample with the ethanol already removed, so you don't have to wait for it to evaporate in real-time.

Allow students to touch each cured material (wearing gloves) and have them record their observations. Small portions of reprocessable networks and thermosets can also be passed around to the class in vials. This is also an opportunity to demonstrate the ability for the reprocessable network to flow over time. If prepared in advance, the reprocessable network should begin to flow and form a continuous shape (Figure S2).

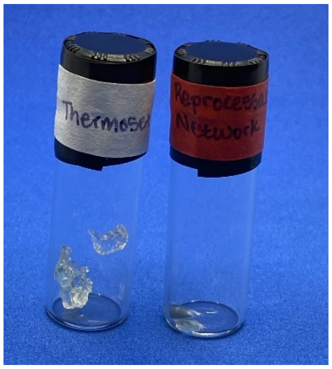

Figure S2. Thermoset and reprocessable network passed around to students. Note: the reprocessable network has formed a raindrop-like shape at the bottom of the vial whereas the thermoset is still the shape as it was when it was added to the vial.

Next the instructor will demonstrate the reprocessability of the reprocessable network and the lack thereof of the thermoset. This process mimics melt-pressing, a technique used frequently in industry and academic settings to create plastic parts with uniform thickness and defined shape. This process is explained in the PowerPoint presentation.

The necessary glassware and equipment for this demonstration are listed below (Figure S3).

- Petri dish
- Tweezers

- Razor blades
- Hot plate capable of reaching 125 °C
- ~500 g weight

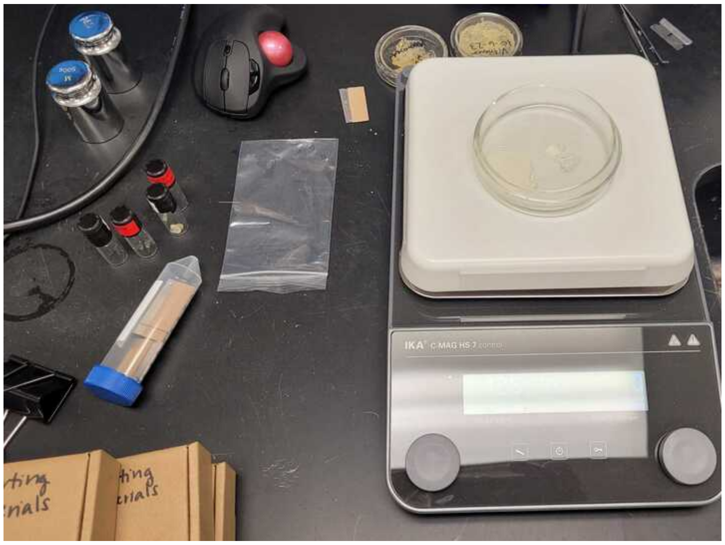

Figure S3. Necessary equipment for reprocessability demonstration.

Procedure for reprocessability demonstration:

1. Preheat a hot plate to 125 °C.
2. Cut 2 pieces (~1.5 cm squares) from the thermoset material. Place one piece on the lid of the petri dish and layer the second piece to overlap the first piece about halfway (see Figure 4 in the main text)
3. Repeat step 1 with the reprocessable network in the same petri dish. Ensure there is sufficient space between the two samples and label each sample.
4. Place the bottom of the evaporation dish over the samples (make sure there is good contact between the two pieces of glass and the networks) and place the dishes on the preheated hot plate.
5. Add the 500 g weight to the top of the petri dish holding the sample and wait ~5-10 minutes. The reprocessable network should reform into one piece, and the thermoset should remain as two individual pieces.
6. Allow students to take turns coming to the hot plate to make observations. What they see should help them answer the concluding questions on the handout.

At the end of the demonstration, let students work in small groups to complete the concluding questions on the handout.

By the end of this second lesson and demonstration, students should be able to:

- List the steps of the scientific method
- Apply the scientific method to the creation of new materials
- Explain what “self-healing” means
- Describe how temperature and pressure affect reprocessable networks and thermosets

EPOXIDATION OF SOYBEAN OIL

If materials or lab space for this synthesis are unavailable for the instructor, collaboration with a local research institution (industrial or academic) may be necessary, and epoxidation should be achievable within a day with low cost. Alternatively, other materials such as epoxidized acrylate soybean oil (ESOA) are available for purchase and while ESOA was not tested in this work, a similar method to what is used in this demonstration should be applicable. The table below summarizes the necessary reagents and amounts. Note: this reaction is scalable to achieve larger quantities.

Table S1. Reactant quantities for soybean oil epoxidation

Reagent	Molecular weight (g/mol)	Mass (g)	Density (g/mL)	Moles	Equivalents
Soybean oil	920	30	0.917	0.0326	1
Meta-chloroperoxybenzoic acid (mCPBA)	176	45.8	-	0.261	8

Additional materials, glassware, and equipment:

- Dichloromethane (DCM) (100 mL)
- 10 wt% sodium hydroxide in water (100 mL)
- 10 wt% brine solution (NaCl in water) (50 mL)
- 500 mL round bottom flask
- 100 mL Erlenmeyer flask
- Ice bath
- Rubber septum (stopper)
- Needle
- Filter paper
- Glass funnel
- 500 mL separatory funnel
- 250 mL beaker
- 250 mL Erlenmeyer flask
- 10 mL syringe
- 0.22 µm PFTE syringe filters

Procedure for preparation of epoxidized soybean oil (ESO):

1. Weigh mCPBA into a 500 mL round bottom flask and dissolve in DCM (~70 mL). Insert a stir bar and mix over ice.
2. Weigh soybean oil into a 100 mL Erlenmeyer flask and dissolve in DCM (~30 mL).
3. Slowly add the contents of the soybean oil solution to the mCPBA flask over ~10 minutes and mix for ~15 minutes before removing the ice. Cover the flask with a stopper and insert a needle to prevent any pressure buildup. Stir at room temperature for 2 hours.
4. Gravity filter the mixture to remove 3-chlorobenzoic acid (byproduct of mCBPA) and add the filtrate to a 500 mL separatory funnel.

5. Add 50 mL of NaOH solution to the separatory funnel to remove remaining mCPBA. Collect the bottom (organic) layer in a 250 mL beaker and discard the top layer. Add the organic layer back to the separatory funnel and repeat step 5.
6. Add 50 mL of brine solution to the separatory funnel. Collect the bottom (organic) layer in a new 250 mL beaker and discard the top layer.
7. Add magnesium sulfate in gram amounts to the beaker until clumping of the solids no longer occurs upon swirling the mixture.
8. Gravity filter the mixture into a 250 mL Erlenmeyer flask. Syringe filter the filtrate from this step into a 500 mL round bottom flask to remove the smaller pieces of magnesium sulfate. Change the filter when necessary.
9. Dry the epoxidized soybean oil to remove the excess DCM. This can be accomplished with a rotary evaporator or under nitrogen/air in a fume hood.

PREPARING REPROCESSABLE NETWORKS AND THERMOSETS (Detailed Instruction)

It is recommended to prepare reprocessable networks and thermosets in advance of class activities for middle school students. These materials should be stored in a desiccator or airtight container to prevent hydrolysis, especially in humid environments. While ESO is a starting material for this reaction, it needs to be made ahead of time by the instructor (see **Epoxidation of Soybean Oil** above).

Necessary materials for preparation of green thermoset and reprocessable network (Figure S4:

- 20 mL scintillation vials
- 8 mL scintillation vials
- Disposable aluminum weighing dishes
- Metal lab spatula
- Hot plate
- Razor blade

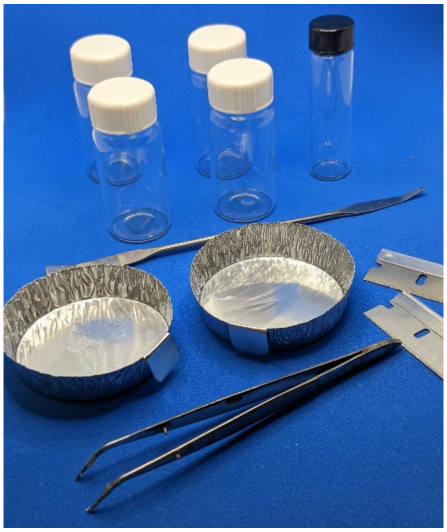

Figure 4. Necessary materials for preparation of thermoset and reprocessable network (excluding hot plate).

For middle school students, a PowerPoint slide illustrates how these materials are made. If there are multiple class periods per day, it is recommended to have a thermoset and reprocessable network sample for each class.

Table S2. Reactant quantities for preparation of the reprocessable network

Reagent	Mass/Volume
ESO	10 g
Citric Acid	2.6 g
Ethanol	8 mL
Baking soda	125.9 mg
Water	1 mL

Procedure for preparation of reprocessable network:

1. In a 20 mL scintillation vial, weigh 10 g of ESO. Add approximately ~2 mL of ethanol to this vial.
2. In a second 20 mL scintillation vial, weigh 2.6 g of citric acid. Add ~6 mL of ethanol to dissolve the citric acid. Note: this may take some time to dissolve and could require shaking, sonication, or gentle heating.
3. In an 8 mL scintillation vial, weigh 125.9 mg of baking soda. Dissolve this in ~1 mL of water. This may require gentle heating.
4. Add the citric acid solution to the soybean oil solution and mix before pouring half of this solution into a labeled disposable aluminum weighing dish.
5. Add the baking soda solution of the aluminum weighing dish. Note: the mixture may become slightly cloudy.
6. Add the remainder of the soybean oil mixture to the aluminum weighing dish and mix the contents of the dish with a spatula until homogenous. Let this dish air dry overnight before transferring to a hot plate.
7. Set the hot plate to 85 °C and ensure the ethanol evaporates from the dish. This should take ~30 minutes.
8. After the ethanol evaporates and there is no more bubbling, set the hot plate to 125 °C and cure for an additional 20 minutes.
9. Once cured, remove the dish from the hot plate and allow to cool to room temperature. The sample can then be removed from the aluminum dish with a razor blade and can be stored in a desiccator or sealed container until the demonstration.

Table S3. Reactant quantities for preparation of the thermoset network

Reagent	Mass/Volume
ESO	10 g
Citric Acid	2.6 g
Ethanol	8 mL

Procedure for preparation of thermoset:

1. In a 20 mL scintillation vial, weigh 10 g of ESO. Add ~2 mL of ethanol to this vial.
2. In a second 20 mL scintillation vial, weigh 2.6 g of citric acid. Add ~6 mL of ethanol to dissolve the citric acid. Note: this may take some time to dissolve and could require shaking, sonication, or gentle heating.
3. Add the citric acid solution to the soybean oil solution and mix before pouring into a labeled disposable aluminum weighing dish. Let this dish air dry overnight before transferring to a hot plate.
4. Set the hot plate to 85 °C and ensure the ethanol evaporates from the dish. This should take approximately 30 minutes.
5. After the ethanol evaporates and there is no more bubbling, set the hot plate to 125 °C and cure for an additional 20 minutes.
6. Once cured, remove the dish from the hot plate and allow to cool to room temperature. The sample can then be removed from the aluminum dish with a razor blade and can be stored in a desiccator or sealed container until the demonstration.

ADDITONAL NOTES

This demonstration can also easily be adapted to a laboratory experiment for older students provided access to sufficient materials for groups of students to make the networks. It is still recommended that the ESO be synthesized in advance as this synthesis is designed for people with knowledge of organic chemistry. The authors recommend adapting this to a high school experiment over 4 class periods with suggestions below:

1. Lecture over introduction to polymers and reprocessable networks. The **Lecture Slides** provided should not require much editing to go from middle school to high school as polymer knowledge is not typically taught in normal curriculum in the US currently.
2. Measure out materials for thermoset and reprocessable network and stop when you get to the point where you leave the samples overnight to allow ethanol to evaporate (see **Preparing Reprocessable Networks and Thermosets** above).
3. Cure both networks.
4. Demonstrate the reprocessability of each material (see **Class 2** above).

For high school students making materials, the **Reprocessable Network Handout** will also need to be adapted to include the information provided in **Preparing Reprocessable Networks and Thermosets**.

In advanced chemistry classes, instructors may introduce infrared spectroscopy as a means of characterizing these materials, but it is not recommended for students in the middle grades. Introductions into functional groups and basic organic chemistry may also be beneficial for advanced high school chemistry.

To ensure the two networks had cured (beyond them no longer being liquid), Fourier transform infrared (FTIR) spectra were collected (Figure S5). FTIR data was collected on a Thermo Fisher Nicolet 6700 FTIR with a Smart iTR ATR sampling accessory and averaged over 32 scans with a 1 cm^{-1} resolution.

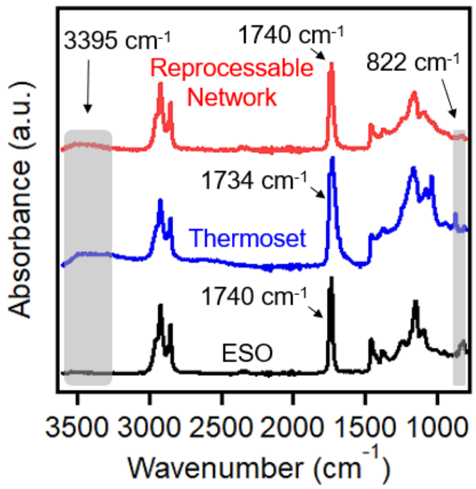

Figure 5. FTIR spectra of ESO, thermoset, and vitrimer.

As the epoxy ring-opening and esterification occur, it is expected to see the appearance of hydroxyl stretching peaks (-OH, weak, broad, ~3200-3400 cm^{-1}), possible shifting of the carbonyl stretching peak (C=O, strong, narrow, ~1650-1800 cm^{-1}) due to the presence of carbonyls with different local environments, and the disappearance of the epoxy group stretching peak (-C-O-C-, weak, narrow, ~750-880 cm^{-1}). The appearance of the hydroxyl stretching peak and disappearance of the epoxy stretching peak in both networks indicate the reaction has occurred.
